# Metatranscriptomics-guided genome-scale metabolic reconstruction reveals the carbon flux and trophic interaction in methanogenic communities

**DOI:** 10.1186/s40168-024-01830-z

**Published:** 2024-07-05

**Authors:** Weifu Yan, Dou Wang, Yubo Wang, Chunxiao Wang, Xi Chen, Lei Liu, Yulin Wang, Yu-You Li, Yoichi Kamagata, Masaru K. Nobu, Tong Zhang

**Affiliations:** 1 Environmental Microbiome Engineering and Biotechnology Laboratory, Center for Environmental Engineering Research, Department of Civil Engineering, The University of Hong Kong, Pokfulam Road, Hong Kong SAR, China; 2 Laboratory of Environmental Protection Engineering, Department of Civil and Environmental Engineering, Graduate School of Engineering, Tohoku University, 6-6-06 Aza-Aoba, Aramaki, Aoba Ward, Sendai, Miyagi 980-8579 Japan; 3 Graduate School of Agriculture, Hokkaido University, Sapporo, 060-8589 Japan; 4 Institute for Extra-Cutting-Edge Science and Technology Avant-Garde Research (X-star), Japan Agency for Marine-Earth Science and Technology (JAMSTEC), Yokosuka, 237-0061 Japan; 5 State Key Laboratory of Marine Pollution, City University of Hong Kong, Hong Kong SAR, China; 6 School of Public Health, The University of Hong Kong, Hong Kong SAR, China; 7 Macau Institute for Applied Research in Medicine and Health, Macau University of Science and Technology, Macao SAR, China

**Keywords:** Anaerobic digestion, Long reads, Omics, Syntrophic bacteria, Reverse electron transfer

## Abstract

**Background:**

Despite rapid advances in genomic-resolved metagenomics and remarkable explosion of metagenome-assembled genomes (MAGs), the function of uncultivated anaerobic lineages and their interactions in carbon mineralization remain largely uncertain, which has profound implications in biotechnology and biogeochemistry.

**Results:**

In this study, we combined long-read sequencing and metatranscriptomics-guided metabolic reconstruction to provide a genome-wide perspective of carbon mineralization flow from polymers to methane in an anaerobic bioreactor. Our results showed that incorporating long reads resulted in a substantial improvement in the quality of metagenomic assemblies, enabling the effective recovery of 132 high-quality genomes meeting stringent criteria of minimum information about a metagenome-assembled genome (MIMAG). In addition, hybrid assembly obtained 51% more prokaryotic genes in comparison to the short-read-only assembly. Metatranscriptomics-guided metabolic reconstruction unveiled the remarkable metabolic flexibility of several novel *Bacteroidales*-affiliated bacteria and populations from *Mesotoga* sp. in scavenging amino acids and sugars. In addition to recovering two circular genomes of previously known but fragmented syntrophic bacteria, two newly identified bacteria within *Syntrophales* were found to be highly engaged in fatty acid oxidation through syntrophic relationships with dominant methanogens *Methanoregulaceae* bin.74 and *Methanothrix* sp. bin.206. The activity of bin.206 preferring acetate as substrate exceeded that of bin.74 with increasing loading, reinforcing the substrate determinantal role.

**Conclusion:**

Overall, our study uncovered some key active anaerobic lineages and their metabolic functions in this complex anaerobic ecosystem, offering a framework for understanding carbon transformations in anaerobic digestion. These findings advance the understanding of metabolic activities and trophic interactions between anaerobic guilds, providing foundational insights into carbon flux within both engineered and natural ecosystems.

Video Abstract

**Supplementary Information:**

The online version contains supplementary material available at 10.1186/s40168-024-01830-z.

## Introduction

Anaerobic digestion is regarded as a representative engineered biotechnology that contributes to creating a circular economy and combatting climate change by organic waste resource recovery and renewable methane production. The global operation of approximately 132,000 digesters (≥ 100 m^3^ tank size) and the growth of biogas-based electricity generation from 64,854 GWh in 2012 to 96,565 GWh in 2020, as reported by the World Gas Association and The International Renewable Energy Agency, highlight the significant global interest of anaerobic digestion biotechnology [1, 2]. Within methanogenic environments, multiple microbial trophic guilds collaborate to convert organic matters into methane and carbon dioxide, playing a pivotal role in anaerobic carbon flux across both natural ecosystems and engineered bioreactors [3]. Despite the widespread application of anaerobic digestion and well-known theories about a cascade of anaerobic degradation steps, knowledge about the uncultured anaerobes, including their genetic diversity and ecological functions, is still relatively scarce not only due to their vast phylogenetic and metabolic diversity but also technical limitations, e.g., challenges in cultivating slow-growing anaerobes [4, 5]. An improved understanding of the metabolic capability of uncultured anaerobes and their adaptations to environmental shifts will contribute to optimizing operational strategies of anaerobic waste treatment and deciphering the black box of anaerobic carbon transformation and the global carbon flux [6].

In recent years, genome-centric metagenomics has extensively been used to explore intricate microbial communities, offering crucial insights into the potential ecological functions of microbial populations within anaerobic environments [3, 7]. However, despite advancements in sequencing technologies and bioinformatic workflows, short-read-based genome-resolved metagenomics is still subject to substantive limitations partly owing to the challenges in reconstructing complete/high-quality genomes and retrieving comprehensive genetic information [8]. Long-read sequencing approaches provide unique solutions for metagenomic assembly challenges by recovering rRNA operons and spanning the long repetitive regions [9, 10], enabling the retrieval of complete and previously unexplored metagenome-assembled genomes (MAGs) in diverse environments, e.g., activated sludge ecosystem [11] and rumen [12]. Several investigations have reported large collections of biogas microbiomes by short-read-based metagenomics. Ma et al. assembled 2426 draft MAGs from 56 full-scale biogas plants in China [13]. And Campanaro et al. retrieved 1401 archaeal and bacterial genomes derived from 134 public metagenomes from various biogas reactors [14], which was further complemented to 4568 non-redundant anaerobic species by integrating 192 additional datasets [15]. However, only 108 MAGs were shared in the collections between Ma et al. and Campanaro et al. [13], highlighting high portion of undiscovered anaerobes inhabiting various methanogenic environments and necessity of recovering the microbial wealth from anaerobic communities by long-read metagenomics.

Given the complicated nature of engineered and natural methanogenic environments, the majority of anaerobic digestion studies focused on a specific facet of the microbial communities, e.g., the methanogenic stage, using simplified model systems [16, 17] or long-term artificial enrichment of substrate specification experiments, such as syntrophic consortia enrichment [6, 18, 19]. Such pioneering studies provided important knowledge about the metabolic function of different flora and their metabolic trophic relationships. However, it is crucial to recognize that both natural and engineered ecosystems consistently harbor a complex mixture of different substrates, rather than simple ones. These substrates collectively determine the ecological niches of microbial populations and significantly influence their metabolic interactions. Therefore, to capture functionally important species and uncover their trophic interactions in complex methanogenic habitats, it is vital to incorporate individual-level functional assignment and community-level carbon mineralization routes. Up to date, few attempts have been made to unravel a genome-wide understanding of carbon flow from sugars and amino acids (AAs) to central carbon metabolism, from syntrophic oxidation of long-chain fatty acids (LCFA) to short-chain fatty acids (SCFA), and finally to methanogenesis within the complex anaerobic food web [20–22]. Furthermore, prior attempts in exploring the metabolic potential of keystone species were mainly based on the presence of targeted pathways or biomarker genes [22–24], with fewer investigations harnessing multi-omics approaches to provide expression-based evidence regarding the in situ metabolic activity of highly active microbes and their biogeochemical functions in intricate ecosystems.

In this study, we investigated in situ metabolic activities and trophic interactions of uncultivated anaerobes by utilizing a lab-scale anaerobic bioreactor as a methanogenic ecosystem. Using metatranscriptomics-guided metabolic reconstruction, we unraveled a community-level carbon flow based on high-quality genomes reconstructed by the hybrid assembly using short and long reads. Through this work, we connected microbial community structures to the functional potentials of individual populations, identified novel keystone lineages with previously undescribed functions, as well as pinpointed specific genomic characteristics of active anaerobic lineages helping them stand out from substrate competition and niche dominance. Our study improves the understanding of metabolic underpinnings and trophic interactions between uncultivated key anaerobic guilds, providing a foundational framework to connect the metabolic attributes of uncultured anaerobic lineages with their ecological function within the intricate anaerobic ecosystems.

## Materials and methods

### Bioreactor operation and sample collection

A lab-scale 15-L anaerobic membrane bioreactor (AnMBR) was inoculated with the mixed anaerobic sludges collected from four full-scale anaerobic tanks, fed with fresh leachate from a refuse transfer station and operated for 215 days at 35 ± 1 °C, achieving a stable and high COD removal efficiency of over 98% (please refer to our previous publication [25] about the detailed operation conditions and the substrate properties).

This AnMBR had been stably operated from long HRT (hydraulic retention time, 20 days) to short HRT (1 day). The organic loading rates changed from low (under HRTs of 20 days, 15 days, and 10 days) to medium (7 days, 5 days, and 4 days), and finally high (3 days, 2 days, and 1 day). To obtain a comprehensive catalog of anaerobic lineages and accurately assess their functional activities, anaerobic digested slurry samples at the seeding phase (namely Raw) and at the time point with the highest methane-produced rates of each HRT (namely H20, H15, H10, H7, H5, H4, H3, H2, and H1) were collected to provide temporal metagenomics and metatranscriptional evidence for a more thorough analysis. All the collected samples were frozen by liquid nitrogen immediately and stored at −80 °C before DNA and RNA extraction.

### DNA and RNA extraction, library construction, and sequencing

Total genomic DNA of anaerobic digested slurry samples was extracted using the DNeasy^®^PowerSoil^®^ Pro Kit (Qiagen, German) in accordance with the manufacturer’s instruction with a slight modification. Specifically, for the cell lysis step, the vortex at maximum speed was shortened to 8 min to avoid over fragmentation of DNA contents. The quantity and quality of the extracted DNA were checked and measured by NanoDrop ONE (Thermo Fisher Scientific, USA) and agarose gel electrophoresis. The extracted DNA was divided into two parts, and the first part was sequenced on the Illumina NovaSeq platform (Illumina, CA, USA) by Novogene Co., Ltd. (Beijing, China) with 2 × 150 bp paired-end strategy while the second part was prepared using the SQK-LSK109 Ligation Sequencing Kit for library construction and sequenced on the GridION X5 platform (Oxford Nanopore Technologies, Oxford, UK) with R9.4.1 flow cell in our lab.

Total RNA from the sludges, taken in three replicates, was extracted using the RNeasy^®^ PowerSoil^®^ Total RNA kit (Qiagen, German) with the addition of phenol: chloroform: isoamyl alcohol (25:24:1). The extracted RNA was checked for integrity and purity using agarose gel electrophoresis and Agilent 2100 Bioanalyzer (Agilent, Santa Clara, CA, USA). Prior to metatranscriptomic sequencing, rRNA was depleted from the total RNA using the Ribo-Zero rRNA removal kit (Illumina, USA), and then the remaining mRNA was fragmented and reverse-transcribed into cDNAs for subsequent sequencing. Illumina sequencing of cDNA samples was performed in Novogene Co., Ltd. (Beijing, China). Detailed statistics of the sequencing data was provided in the Supplementary Data 1.

### Data quality control, metagenomic assembly, and binning

For Illumina sequencing data, fastp (v0.23.2) [26] was used to filter out low-quality and contaminated reads with the parameters “–cut_mean_ quality 20 --detect_adapter_for_pe.” For Nanopore sequencing data, base calling was performed by Guppy (v5.0.11). Quality control of the basecalled reads was conducted using NanoPlot (v1.40.2) [27] with a quality threshold of *Q* ≥ 7. Only reads longer than 1000 bp were used for downstream analysis.

Four approaches with different assembled strategies were used to assemble the quality-controlled short and long reads to obtain high-quality contigs: two short-read-based assemblers, metaSPAdes (v3.14.1) [28] and megahit (v1.2.9) [29], and two hybrid methods, Unicycler (v0.4.4) [30] and iterative haplotype-resolved hierarchical clustering-based hybrid assembly approach [31]. Details about the assembly approaches are provided in Supplementary Methods. The assembled contigs of each approach were imported into MetaWRAP (v1.3.2) [32] separately for metagenomic binning. The bin_refinement module from MetaWRAP was used to consolidate the binning results from MaxBin2 [33], metaBAT2 [34], and CONCOCT [35] to recover a single and improved bin set. The refined bins from each assembly approach were aggregated and dereplicated using dRep (v3.2.2) [36] at the 99% (strain level; Supplementary Data 2) and 95% (species level; Supplementary Data 3) average nucleotide identity (ANI), respectively. The dereplicated bins were then manually checked to select the representative MAGs with high contiguity.

The quality of the recovered MAGs was evaluated using CheckM (v1.1.3) [37] based on the presence of lineage-specific single-copy marker genes with default parameters. The taxonomy of MAGs was classified based on the Genome Taxonomy DataBase (GTDB) (214 version) using GTDB-Tk (v2.2.6) with the “classify_wf” workflow [38, 39]. The relative abundances of the MAGs were calculated using CoverM (v0.6.1) (https://github.com/wwood/CoverM) in “genome” mode with a minimum identity of 95% and a minimum aligned length of 75%.

### Genome annotation, pathway curation, and metabolic reconstruction

The genome features of the recovered MAGs, including 5S, 16S, 23S rRNA operons, and tRNA were predicted by Prokka (v1.13) [40] with the mode “--kingdom Bacteria” and “--kingdom Archaea,” respectively. Open Reading Frames (ORFs) were predicted using Prodigal (v2.6.3) [41] with the mode “-meta” and the completeness of each ORF was identified by the indicator “partial” in the gene coordinates file. The ORFs of each MAG labeled with the MAG name were merged to create an entire ORF catalog for metatranscriptomic analysis. The ORF catalog was functionally annotated with Kyoto Encyclopedia of Genes and Genome (KEGG) orthologous group ids (KO) using online GhostKOALA with the KEGG GENES database of “genus_prokaryotes” option [42, 43].

A total of 90 anaerobic pathways are summarized, elaborating the carbon flux from sugars and AAs to central carbohydrate metabolism, from LCFA to SCFA oxidation, and finally to methanogenesis (Supplementary Data 4). To ensure the accuracy of genes associated with a specific pathway, KOs in each step’s reactions were manually checked in the KEGG and MetaCyc databases [43, 44]. Due to the lack of modules for some pathways, the modules were constructed based on rules referred to the KEGG existing modules (Supplementary Methods). To accurately assign the metabolic abilities with a MAG, a specific pathway is considered to exist in a MAG only if 100% of its key reactions were identified within that MAG, with the exception of the methylmalonyl-CoA pathway (MMC) for syntrophic propionate oxidation. Considering the imperfection in MAGs and 11 steps involved in this pathway, the presence of MMC pathway in a MAG was defined as the identification of ≥ 90% of key steps (10 of 11 steps).

### Genome-centric metatranscriptomic analyses

After quality control, SortMeRNA (v4.3.4) [45] was used to remove non-coding RNA sequences from metatranscriptomic reads according to the multiple rRNA databases for bacterial, archaeal, and eukaryotic sequences. Resulting mRNA reads were mapped to the entire ORF catalog collected from all the recovered MAGs using RSEM (v1.3.3) [46] to calculate the read count of each ORF. To make a comparison between samples, the unit of transcripts per million (TPM) was employed to quantify the transcriptional expression level of each gene, which were normalized by the length of each gene and the sequencing depth per sample [47].

To identify the active populations at different stages of carbon mineralization, we calculated the transcriptional expression of targeted pathway in each MAG and conducted a comparative analysis of transcriptional activities within a specific pathway between different MAGs. The transcriptional expression of targeted pathway in one MAG was calculated as the average transcriptional expression of all the steps. Moreover, the transcriptional expression level of a particular step was determined through the following criteria: (1) if a step could be catalyzed by a single enzyme complex, the average transcriptional expression of each subunit was employed; (2) if a reaction could be catalyzed by multiple enzymes or multiple copies of an enzyme encoded by a genome, their summed transcriptional expression was utilized as the expression value of that step [47]. The overall analysis workflow is shown in Supplementary Fig. 1, and additional methodological details are provided in Supplementary Methods.

## Results

### Hybrid assembly improves the quality of metagenome-assembled genomes

After 215-day operation of the anaerobic reactor, 10 anaerobic digested slurry samples under increased organic loadings from HRT of 20 to 1 day were sampled for sequencing. In total, we obtained 109 Gb Illumina short-read data (average 10.9 Gb per sample) and 58 Gb Nanopore long-read data (average 14.5 Gb per sample) (Supplementary Data 1). Then short-read-only assembly and hybrid assembly strategies were utilized, followed by a comprehensive assessment of assembly contiguity, genetic information recovery, and MAG quality.

**Fig. 1:**
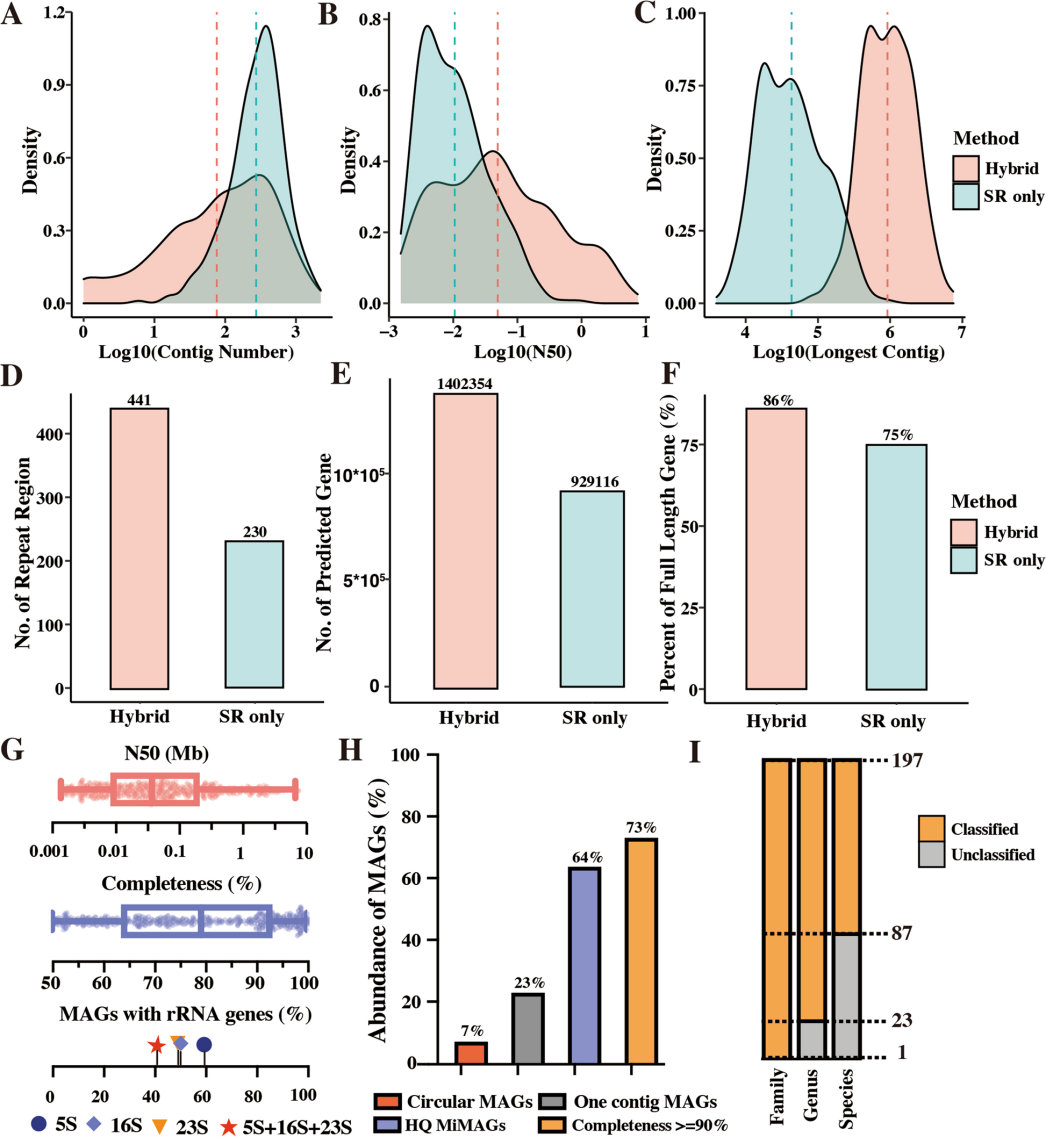
Long-read sequencing improved metagenomic assembly and recovered hidden genetic information in the methanogenic community. **A–C** The log-scale distribution of contig number and lengths for short-reads-only approach (SR-only) and hybrid assembly (Hybrid). The dashed lines indicate median values. **D–F** The recovery of repeat regions and predicted genes as well as the proportion of full-length genes in the assembled metagenome-assembled genomes (MAGs) by short-reads-based method (SR-only) and hybrid assembly (Hybrid). **G** The genomic information of MAG at strain-level by hybrid assembly. **H** The cumulative relative abundances of high-quality genomes in the anaerobic community. **I** Unclassified MAGs at different taxonomic levels by GTDB (214 version)

The comparison results showed that hybrid assembly significantly improved the contiguity of assemblies, having fewer contig counts in a MAG (averagely 241 vs 397) and remarkably longer (13 times) N50 length (averagely 342.37 kbp) (Fig. 1A–C, Supplementary Data 5). In addition, it has been demonstrated that long reads could considerably span the challenging regions, such as rRNA operons and repetitive regions. In this study, 366 full-length 16S rRNA genes were identified in 243 MAGs (45% of the total MAGs) by hybrid assembly, while the short-read-based assembly only recovered 15 full-length 16S rRNA genes (Supplementary Data 5). For repetitive regions, 441 in 204 MAGs were obtained with hybrid assembly while only 230 using short-read assembly (Fig. 1D). Regarding predicted genes, hybrid assembly not only retrieved 51% more prokaryotic genes (1,402,354 vs 929,116) but also possessed a higher proportion (86% vs 75%) of full-length prokaryotic genes compared to the short-read-only assembly (Fig. 1E, F). Following the stringent criteria of MIMAG [48], 132 of the reconstructed MAGs using the hybrid assembly were designated as high-quality genomes, much more than that (only 3 MAGs) from the short-read-based assembly (Supplementary Data 5). Of the eight assembled circular MAGs, three were proposed as potential a new genus, a new family, and a new order based on their full-length 16S rRNA gene identity (Supplementary Note 2, Supplementary Data 6). Overall, hybrid assembly plays a crucial role in improving the genome quality of MAGs, which will largely facilitate the exploration of complete metabolic processes by capturing the genetic information missed in the short-read-only assembly.

### High-quality MAGs represent the majority of methanogenic community

After dereplication at 99% ANI threshold, a total of 542 MAGs with a median N50 length of 104 kbp and median completeness of 79.2% were recovered (Fig. 1G). By using 95% ANI and further quality filtration, there were 197 species-level MAGs with a completeness of ≥ 90%, among them 132 were high-quality genomes, and 20 out of the 132 genomes with one contig being nearly complete (Supplementary Data 3). Remarkably, the abundances of high-quality genomes accounted for ~64% of anaerobic microbiomes in the reactor at HRT of 1 day (Fig. 1H, Supplementary Data 3), indicating that these high-quality MAGs could provide a high characterization of encoded metabolic potentials and hidden genomic features in the methanogenic community of this study.

**Fig. 2:**
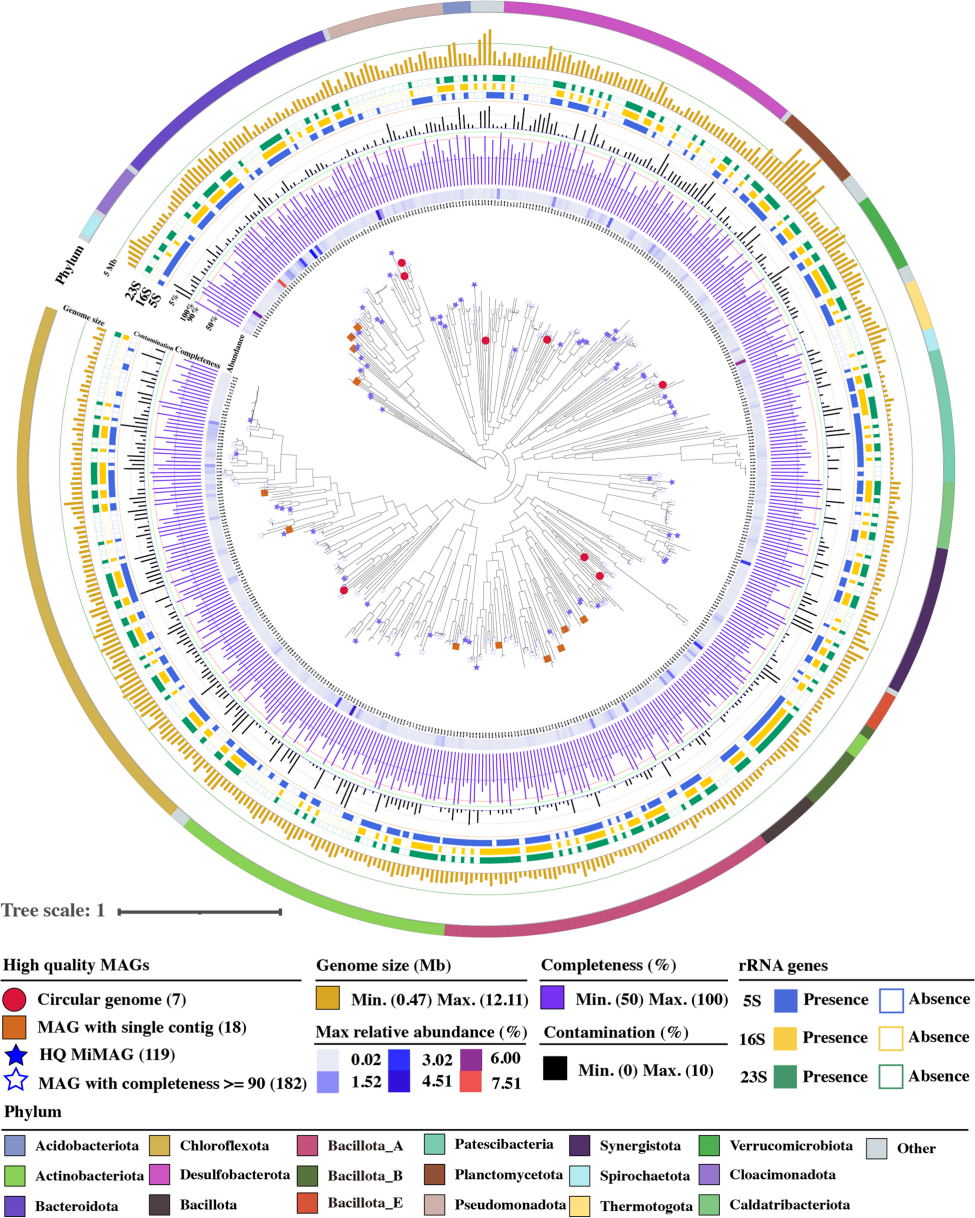
Phylogenetic genome tree showing the diversity, abundance, and genome quality of the bacterial lineages. The tree is constructed based on the concatenated alignment of 120 single-copy bacterial-specific marker genes by GTDB-Tk. Outer bands with different colors show the phyla of all the bacterial lineages. Inner heatmap indicates the maximum relative abundance of the MAGs in different sampling points. Bar charts and heatmap strips from the inner to the outer sections indicate the genome quality of each MAG. Other detailed information is provided in Supplementary Data 3

Phylogenetic analyses revealed a broad taxonomic diversity of the 197 genomes, encompassing four archaeal (15 MAGs) and 23 bacterial phyla (182) (Fig. 2, Supplementary Data 3). And the majority of 197 MAGs were affiliated with the *Chloroflexota* (30), *Bacillota*_A (25), *Bacteroidota* (25), *Acidobacteriota* (20), and *Desulfobacterota* (18). Of note, 87 of the 197 MAGs (44.16%) did not match any of the reference species genomes in the GTDB (214 version), implying that they belong to unknown populations at the species level or higher taxa (Fig. 1I, Supplementary Data 6). We also performed a species-level comparison of 197 MAGs to two public large sets of anaerobic MAGs from full-scale biogas plants and reactors [13, 14], unveiling the significant MAGs uniqueness (65.5%) in our study and a substantial improvement in quality (94.1%) for the aligned 68 MAGs in previous public dataset (Supplementary Note, Supplementary Data 6). This result emphasized the vast phylogenetic and metabolic traits of unexplored anaerobic lineages in diverse intricate ecosystems, warranting further investigation.

### Metatranscriptomic and metabolic reconstruction of anaerobic community

The 197 MAGs showed good representativeness of the active populations in the anaerobic community, with 81.4% ± 1.9% of the metatranscriptomic reads in each experimental condition mapping to them (Supplementary Fig. 2, Supplementary Data 3). Notably, a significant shift in transcriptional expression was observed from bacterial to archaeal clades as substrate concentrations increased, highlighting an intriguing pattern of gene expression dynamics (Supplementary Fig. 3). At low substrate concentrations (HRT=15 days), approximately 75% of the mapped transcriptomes was attributed to the bacterial clades, with *Mesotoga* cluster (20.3% bin.190 and 1.4% bin.512) accounting for 21.7%, followed by *Bacteroidales*-related cluster at 48.3% (17.4% bin.334, 13.1% bin.202, and 12.3% bin.267) and *Cloacimonadaceae*-affiliated bin.480 at 2.1%. In contrast, under HRT of 2 days, *Methanoregulaceae* bin.74 accounted for the highest proportion (15.8%) of the mapped transcriptomes, followed by two *Methanothrix*-affiliated species bin.206 and bin.266 (13.9%) and *Desulfobacterota* clade (11.4%), mainly including *Smithellaceae* bin.332 (6.9%), *Syntrophobacteraceae* bin.487 (2.7%), and *Syntrophales* bin.292 (1.8%) (Supplementary Fig. 4).

**Fig. 3:**
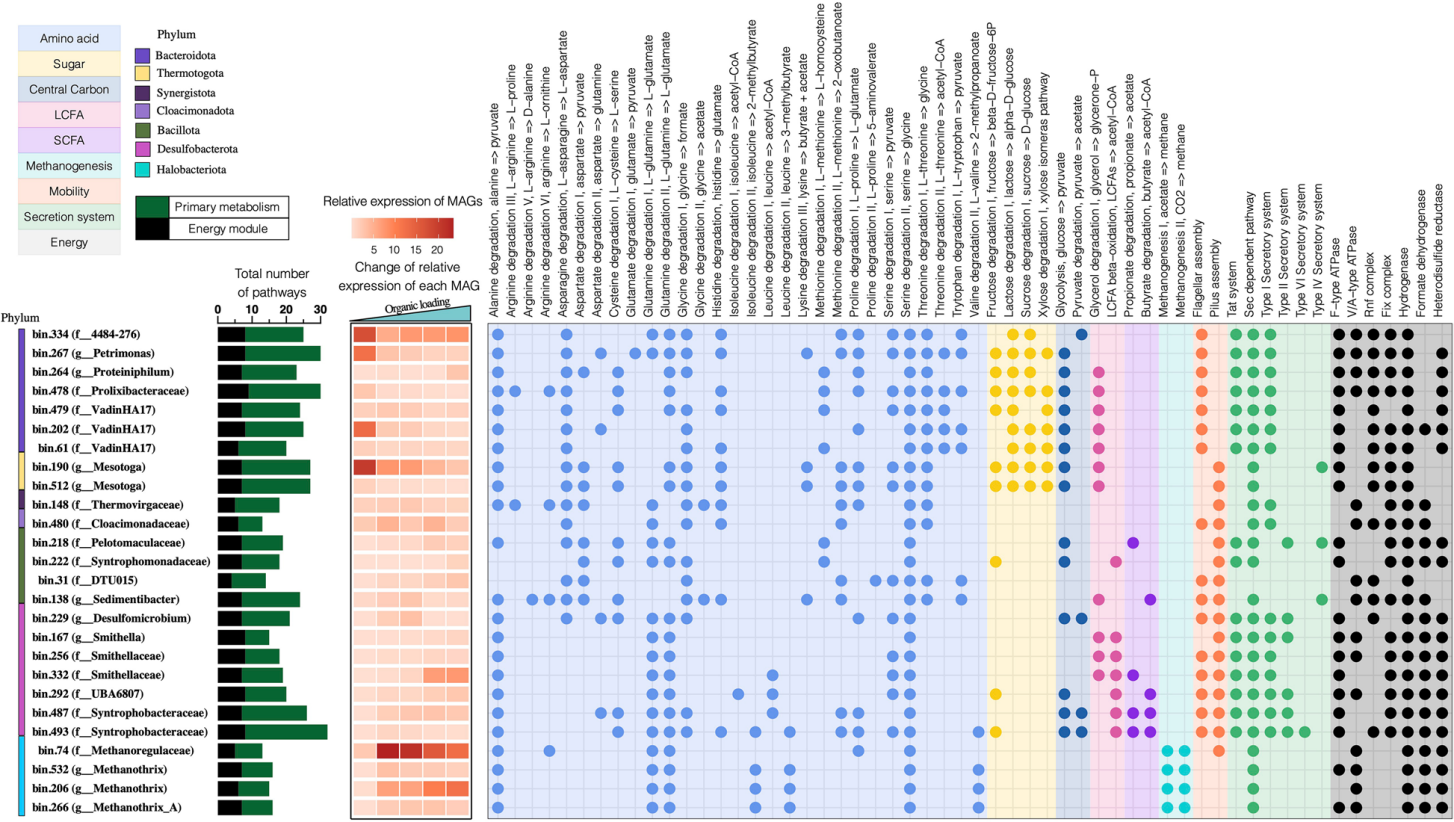
The distribution of metabolic pathways and profiles of functional systems in the top 26 species representatives with a relative transcriptional expression of over 1% in at least one sample of metatranscriptomics. Pathways are considered present only if 100% of steps were identified in a MAG (except 90% for methylmalonyl-CoA pathway). Bar chart demonstrates the number of MAGs encoding the metabolic pathways and energy modules. Heatmap indicates the variation of relative transcriptional expression of each MAG across increased organic loadings

**Fig. 4:**
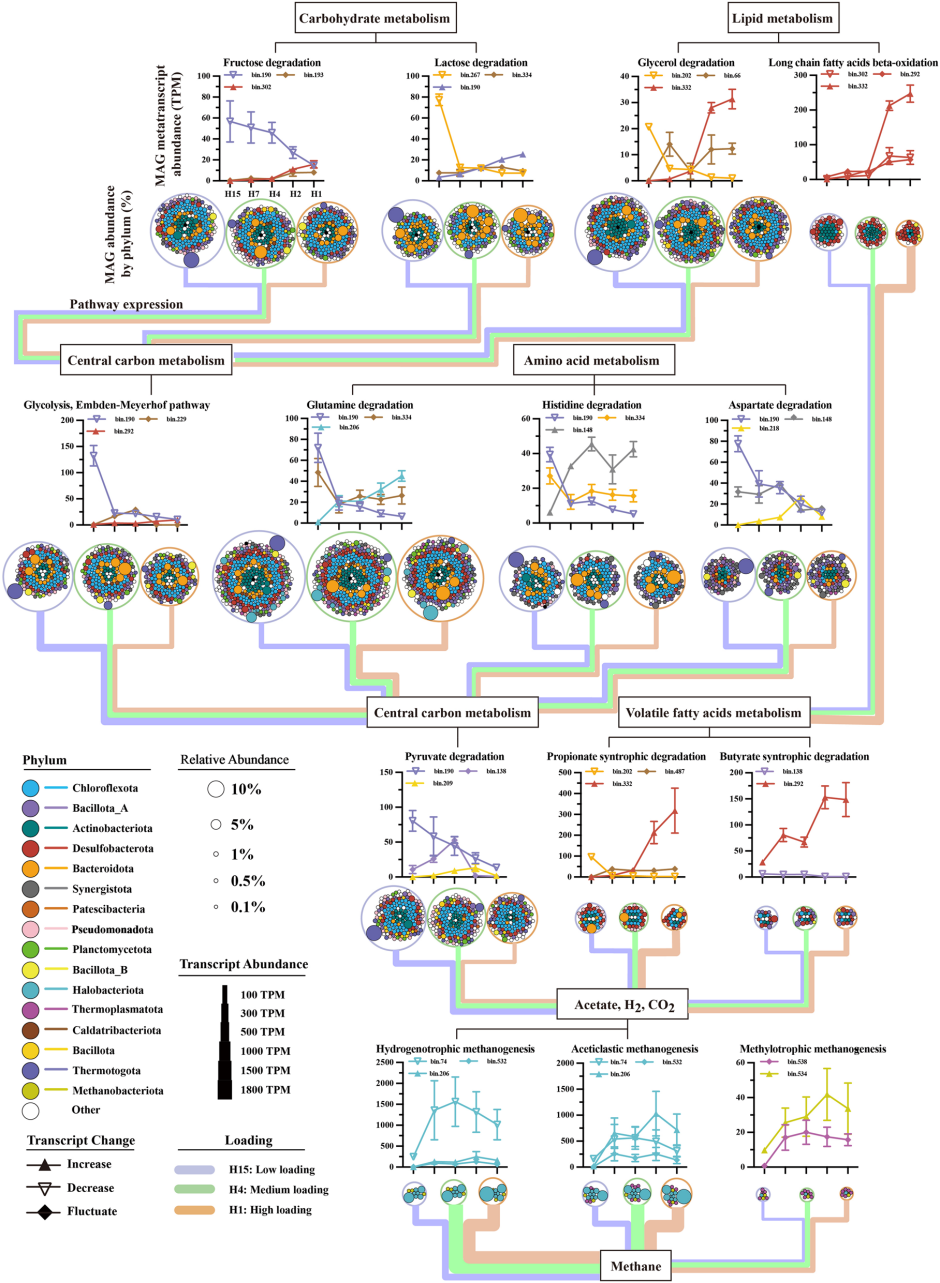
Carbon flux from (poly) monomers to CH_4_ and CO_2_ across the organic loading gradient. Headers in the boxes and the line chart represent degradation modules and specific pathways, respectively. Line chart shows the variations in transcriptional activity of anaerobic populations responding to organic loadings. The lines colors represent the species in different phyla. The large circles under line charts colored by organic loadings encompass smaller circles (MAG abundance colored by phylum) represent MAGs encoding the targeted pathways. Circle size indicates MAG relative abundance. Thickness of lines connecting different modules represents the summed transcriptional expression of specific pathways in different MAGs

By further metabolic reconstruction, we connected each anaerobic lineage with their catabolic abilities, unveiling versatile metabolic potentials of the whole anaerobic microbiota in assimilating carbohydrates, proteins, and LCFAs (Fig. 3, Supplementary Data 7). To further confirm substrate-assimilating function, we provide multiple HRT-dependent transcriptional evidence to identify novel microorganisms and their specific pathways as well as microbes with previously uncharacterized functions. Based on community-level metabolic reconstruction, the genome-wide carbon flux was depicted to track the biotransformation from (poly)monomers to CH_4_ and CO_2_ along the organic loading gradients (Fig. 4). Moreover, we conducted a comparative analysis of transcriptional activities within a specific pathway between different MAGs to identify the active populations at different stages of carbon mineralization (Supplementary Data 8). The sections below focused on catabolic pathways, substrate transport, and energy conservation strategies of the representative active species-level populations within their respective anaerobic guilds.

### Carbohydrate depolymerization and sugar degradation

Consistent with the carbohydrate removal rates of over 98% (Supplementary Fig. 5), the anaerobic microbiota expressed a wide group of enzymes for the carbohydrate depolymerization (Supplementary Data 9). Specifically, 129 glycoside hydrolase (GH, EC 3.2.1.-) families were identified, some of which displayed high transcriptional expression responding to various organic loadings, such as GH13 and GH23 involved in the degradation of starch and peptidoglycan (Supplementary Fig. 6, Supplementary Data 9). Taxonomically, the members of phylum *Bacteroidota* were the dominant contributors (28.5 ~ 67.6%) of the transcriptionally expressed genes encoding carbohydrate-active enzymes (CAZymes) across all conditions, in accordance with prior metagenomic studies highlighting their potential metabolic capacities for carbohydrate utilization [49].

Transcriptional analysis revealed the most active populations in CAZymes for *Bacteroidota* as three uncultured family-level lineages: *Petrimonas* sp. bin.267 (circular genome, 98.9% completeness), *Bacteroidales* bin.334 (contig=1, 95.4% completeness), and *VadinHA17* bin.202 (contig=1, 93.0% completeness), and for *Thermotogota*, *Mesotoga* sp. bin.190 (contig=49, 99.8% completeness) (Supplementary Fig. 4). Transcriptomics-based metabolic reconstruction revealed their similar metabolic utilization of disaccharide (lactose and sucrose), monosaccharide (xylose) and central carbon metabolism, i.e., glycogen degradation, glycolysis, and/or pyruvate oxidation, confirming the overlapped functional traits within the community (Fig. 3, Supplementary Data 10). However, the dominant pathway activities within genomes indicated the function-specific differences between these populations. Specifically, *Mesotoga* sp. bin.190 had higher transcriptional level of fructokinase to degrade fructose, whereas *Petrimonas* sp. bin.267 expressed more beta-galactosidase (*lacZ*) homologs for lactose cleavage, and *VadinHA17* bin.202 showed higher transcriptional expression level of genes (*xylA* and *xylB*) in xylose isomerase pathway. In addition, these three populations were highly engaged in glycolysis to convert glucose into pyruvate, while *Bacteroidales* bin.334 showed a preference for downstream pyruvate oxidation to produce acetate, supported by its absence of the complete glycolysis pathway and higher transcriptional expression of pyruvate:ferredoxin oxidoreductase (PFOR), phosphate acetyltransferase (*pta*), and acetate kinase (*ack*) (Supplementary Data 10). These results suggested that bacteria with a similar metabolic repertoire exhibit substrate preference to avoid excessive competition and foster interactions within the community. Furthermore, these active taxa utilized different sugar transportation to create specific functional niches. For example, *Thermotogota*-related bin.190 highly transcriptionally expressed gene (*glcEFH*) encoding ABC-type transporter for glucose uptake, whereas *Bacteroidota*-affiliated bin.267 utilized the electrochemical potential-driven transporter (*glcU*) (Supplementary Data 10), indicating the distinct functional adaptations for substrate competition among different taxonomic lineages.

### Protein hydrolysis and amino acid degradation

The high removal efficiencies of proteins (over 98%, Supplementary Fig. S5) suggested that protein and its subsequent hydrolysate amino acids (AAs) were one of the major organic carbon sources within this anaerobic food web. In agreement with this, microbial community members transcriptionally expressing a wide range of peptidases involved in the breakdown of peptide bonds were observed, some of which were predicted to be extracellular (Supplementary Data 11). Notably, *Bacteroidota*-affiliated bin.334 and bin.267, along with *Thermotogota*-affiliated bin.190 and *Thermovirgaceae* bin.148 (contig=8, 100% completeness) showed high proteolytic activity, as evidenced by the high transcriptional expression of genes encoding extracellular and/or intracellular cysteine, serine peptidases, and metallopeptidases in their genomes (Supplementary Data 11). This result is in accordance with findings from 16S rRNA gene sequencing and omics-based method [50, 51], reinforcing the ecological significance of *Bacteroidota*, *Thermotogota*, and *Synergistota* members in protein hydrolysis and AA degradation. Furthermore, *Bacteroidota*-affiliated bin.334 and bin.267, *Thermotogota*-affiliated bin.190 and *Thermovirgaceae* bin.148 encoded and expressed 12, 16, 13, and 13 pathways for the intracellular AAs breakdown, respectively (Fig. 3). Specifically, bin.190 and bin.148 also transcriptionally expressed numerous AAs transporters, i.e., ABC-type transporter for polar and branched-chain AAs, tryptophan/tyrosine transportation, and glycine betaine/proline transport system (Supplementary Data 11).

In addition, a recent study revealed the previously underestimated but crucial role of uncharacterized family, i.e., *VadinHA17*, in the AA degradation [52]. Our study found that two *VadinHA17*-affiliated genomes, bin.479 (contig=9, 97.1% completeness) and bin.61 (contig=150, 93.1% completeness), exhibited ANI values over 98% with two previously identified AA degraders [52] (Supplementary Data 6). The two populations in this study encoded several AA degradation pathways and dipeptide/tripeptide transporters, in line with their previously known AA degradation capabilities. Yet, the pathways responsible for AA degradation exhibited relatively low transcriptional expressions in these two genomes, indicating the limited involvement of these known AA degraders in AA degradation in this study (Supplementary Data 10). By contrast, another *VadinHA17*-affiliated lineage bin.202 (contig=1, 93.0% completeness) that shared a low ANI of 79.1% with those known AA degraders exhibited high abundance (5.2% of total abundance) and transcriptional expressions (13.0 % of total metatranscriptome) in AA degradation under HRT of 7d (Supplementary Fig. 4). Genomic feature analysis has revealed that this previously undescribed genome transcriptionally expressed genes encoding 190 peptidase and 9 intracellular AA degradation pathways for alanine, asparagine, aspartate, glycine, proline, serine, threonine, and tryptophan, as well as transporters, i.e., sodium ion:proline symporter (10), proton/sodium ion:glutamate/aspartate symporter (2), and lipoprotein transporters (9) (Supplementary Data 10 and Data 11). This finding revealed the novel species in AA degradation and implied that metabolic versatility and diversity of family *VadinHA17* remain understudied, particularly with regard to proteolytic activity and AA-scavenging functions.

### Novel bacteria for long-chain fatty acid degradation

The substantial increase in transcriptional level observed in the LCFA pathways with the increased organic loadings (Fig. 4) indicated the presence of potential LCFA degraders within this anaerobic microbiota. A total of 64 MAGs was proposed as the potential LCFA degraders via the complete beta-oxidization pathway, including members of *Desulfobacterota*, *Pseudomonadota* (previously known as *Proteobacteria*), and *Bacillota* (formerly known as *Firmicutes*) (Fig. 4), consistent with results from studies on the anaerobic LCFA-degrading communities based on 16S rRNA gene or metagenomic sequencing [53–56]. Specifically, two populations in family *Smithellaceae*: bin.332 (contig=26, 92.9% completeness) and bin.167 (contig=26, 96.1% completeness), and one *Syntrophales* population, bin.292 (contig=13, 90.3% completeness), were identified as the key active LCFA-degraders, as strongly evidenced by their increased activities and relatively high transcriptional expression (42.1 ± 8.8%, 9.6 ± 0.7%, and 9.1 ± 1.4%) in the beta-oxidation pathway under the highest organic loading (Supplementary Data 8 and Data 12). This finding further provided transcriptomic evidence for the vital role of family *Smithellaceae* in LCFA breakdown [56]. Moreover, *Smithellaceae* bin.332 and *Syntrophales* bin.292 were recognized as the novel LCFA-degrading bacteria (discussed in Supplementary Note 6.2), highlighting the incomplete characterization of microbial genomes involved in LCFA breakdown and the importance of omics-based approaches in discovering novel anaerobic microbes with unidentified functions from complex environments.

**Fig. 5:**
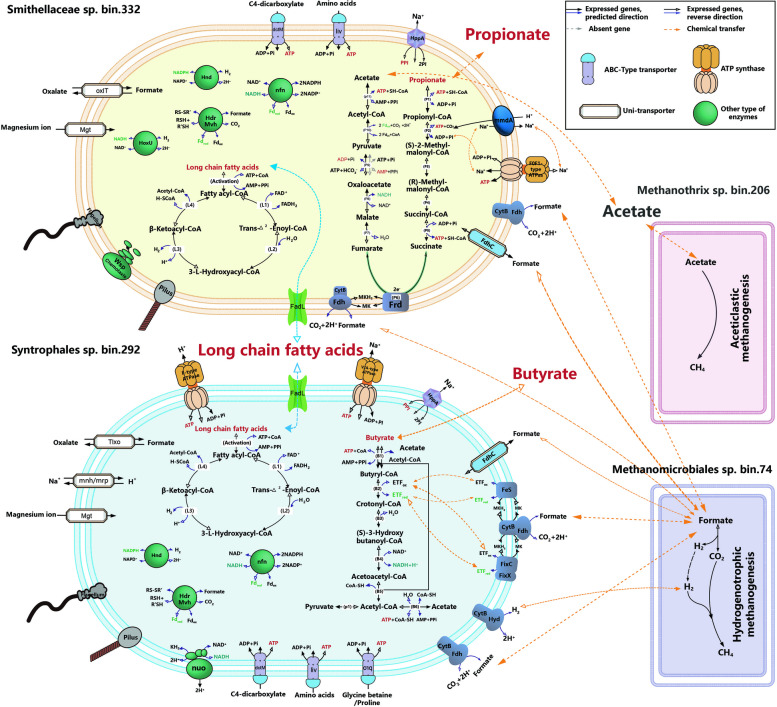
Metabolic reconstruction in the key novel syntrophic fatty acid-oxidizing bacteria and their interactions with the most active methanogens. Only the long- and short-chain fatty acid oxidation pathways, the reverse electron transfer mechanism and transporters are presented in the syntrophic bacteria. Light pink and violet cell cartoons present two predominant methanogens of *Methanothrix* (bin.206) preferring acetate and *Methanoregulaceae* (bin.74) consuming H_2_ and formate generated by the bacteria. The other detailed information of pathway reconstructions is provided in Supplementary Data S12

The above three key active LCFA-degrading bacteria harbored and/or transcriptionally expressed gene encoding a protein FadL to transport fatty acids into the bacterial cytoplasm [57]. And the transported fatty acids were activated by high transcriptional expression of the gene (*ACSL, fadD*) encoding long-chain acyl-CoA synthetase to form fatty acyl-CoA. Subsequently, the fatty acyl-CoA undergoes repetitive beta-oxidation cycles, which is facilitated by the high transcriptional expression of specific enzymes encoded by acyl-CoA dehydrogenase (*acd*), enoyl-CoA hydratase (*echA*), 3L-hydroxyacyl-CoA dehydrogenase (*fadN*), and acetyl-CoA acyltransferase (*fadI*), especially in bin.332 and bin.292 (Fig. 5, Supplementary Data 12). Interestingly, in addition to the LCFA beta-oxidation pathway, bin.332 and bin.292 also exhibited high transcriptional expression of propionate oxidation and butyrate beta-oxidation pathway, respectively. Similar metabolic capacity for simultaneous degradation of LCFA and SCFA has been observed in isolates within the genus *Syntrophomonas*, e.g., *Syntrophomonas palmitatica* strain MPA and *Syntrophomonas zehnderi* strain OL-4 [58, 59]. These findings indicated that these syntrophic populations possessed metabolic flexibility, enabling them to utilize fatty acids of different lengths and saturation levels for growth and energy production.

### Short-chain fatty acid syntrophic degradation

Non-accumulation of SCFAs in effluent relative to their high concentrations in substrate implied the vital contribution of syntrophic SCFA oxidation to the substrate conversion. Despite the high amount of acetate in the substrate and its production during the LCFA breakdown, acetate conversion in this reactor is primarily driven by aceticlastic methanogens rather than bacterial syntrophic oxidation, as elaborated in the Supplementary Note5.

Reconstructing the MMC pathway for propionate oxidation and integrating it with transcriptional activities pinpointed *Smithellaceae* bin.332 (contig=26, 92.9% completeness), *Syntrophobacteraceae* bin.487 (circular genome, 99.5% completeness), and *Pelotomaculaceae* bin.218 (circular genome, 100% completeness) as prominent active participants in propionate degradation (Fig. 4). Bin.487 and bin.218 showed high ANI similarities with the previously characterized syntrophic propionate-oxidizing bacteria (SPOB) [60, 61] (Supplementary Note 6.1), confirming their propionate degradation capabilities and validating the reliability of assembly, binning strategies, and metabolic reconstruction in this study. Remarkably, the two previously identified SPOB had either only 16S rRNA gene or highly fragmented genome (contig=404, 74.7% completeness) [60, 61], underscoring the substantial progress by retrieving circular genomes in our study to better understand their role in propionate metabolism at the genomic level. Of greater significance, the most active population *Smithellaceae* bin.332 was recognized as a novel SPOB (Supplementary Note 6.2), broadening our understanding beyond the previously recognized *Smithella propionica* as the sole SPOB within the *Smithella* genus [19].

Further genomic features and a comparative analysis of transcriptional activity for key genes unveiled unique mechanisms and specific enzymes utilized by these SPOB in energy-dependent processes in the MMC pathway (Fig. 5, Supplementary Data 12). To activate propionate, SPOB could encode a CoA transferase to couple this step (Step1) with the downstream exergonic acetyl-CoA dethiolation (Step11), and/or utilized acyl-CoA synthetases to independently catalyze this step [18, 19, 61]. For *Smithellaceae* bin.322, it harbors four homologs of acyl-CoA synthetases to produce propionyl-CoA, with one (bin332_5_143) showing notably high transcriptional expression, implying that this population decoupled propionate activation from acetyl-CoA hydrolysis without conserving energy. The same activating reaction was also observed in *Pelotomaculaceae* bin.218 and *Syntrophobacteraceae* bin.487 (Supplementary Data 12). For the subsequent carboxylation of propionyl-CoA, unlike other well-described SPOB that could couple this step with downstream decarboxylation of oxaloacetate (Step 9) [19], these three SPOB only harbored and highly transcriptionally expressed gene encoding membrane-associated protein methylmalonyl-CoA decarboxylase (EC 7.2.4.3, bin332_1_53, bin487_1_100 and bin218_1_934), signifying their independent utilization of methylmalonyl-CoA decarboxylase to generate a sodium ion motive force for ATP synthesis and energy conservation [62]. For the energy-dependent succinate oxidation (Step 6), *Syntrophobacteraceae* bin.487 and *Pelotomaculaceae* bin.218 possess two fumarate reductase/succinate dehydrogenase complexes (frdABC: bin487_1_2317 to bin487_1_2319 and bin218_1_611 to bin218_1_613; sdhAB: bin487_1_1345 and 1346, bin218_1_289 and 290) to complete this process (only one in *Smithellaceae* bin.332 probably due to genome incompleteness). Upon further transcriptional activity comparison of these complexes, the consistently high expression of frdABC in *Syntrophobacteraceae* bin.487 and *Pelotomaculaceae* bin.218 implies the more crucial importance of frdABC in this energetically challenging step within the MMC pathway.

Butyrate beta-oxidation pathway reconstruction combined with the transcriptional activity found that only *Syntrophales* bin.292 transcriptionally expressed all the genes involved in the beta-oxidizing pathway, probably due to the rigorous pathway filtering criteria (100% completeness). *Syntrophales* bin.292 expressed all four types of flavin-based electron bifurcation/confurcation systems and several mechanisms facilitating interspecies electron transfer, i.e., hydrogenases and formate dehydrogenases (Supplementary Data 12). A unique characteristic of syntrophic butyrate metabolism is the requirement for reverse electron transport to convert butyryl-CoA into crotonyl-CoA, which could be catalyzed via the membrane-bound complexes (i.e., iron-sulfur-binding reductase and Fix system) as well-described in *S. wolfei* and *S. aciditrophicus* [63, 64]. *Syntrophomonas* bin.292 encodes the complete Fix system (bin292_1_900 to bin292_1_904) and the Fe-S oxidoreductase-EtfAB complex (bin292_2_157 to bin292_2_159), both of which are co-clustered with the butyryl-CoA dehydrogenase (*bcd*) (Supplementary Data 12). Both ETF-related complexes were expressed, yet the Fe-S-binding complex exhibited significantly higher transcriptional activity, underscoring its greater significance in facilitating reverse electron transport during butyrate metabolism. Interestingly, the iron-sulfur-binding reductase was also clustered with genes encoding LCFA transporters (*fadL*), synthetase (K01897 and K01895), NAD (P)H dehydrogenase complexes (*wrbA*), and LCFA-degrading enzymes, such as hydratase and acetyltransferase, forming a concerted gene cluster (bin292_2_146 to bin292_2_160) for fatty acid transportation, activation, and subsequent metabolism (Supplementary Data 12). The presence of these specific genomic features suggests a synergistic role among key functional genes, contributing to the growth of *Syntrophales* bin.292 by utilizing various types and concentrations of fatty acids as substrates in bioreactor.

### Archaea competition in methanogenesis

**Fig. 6:**
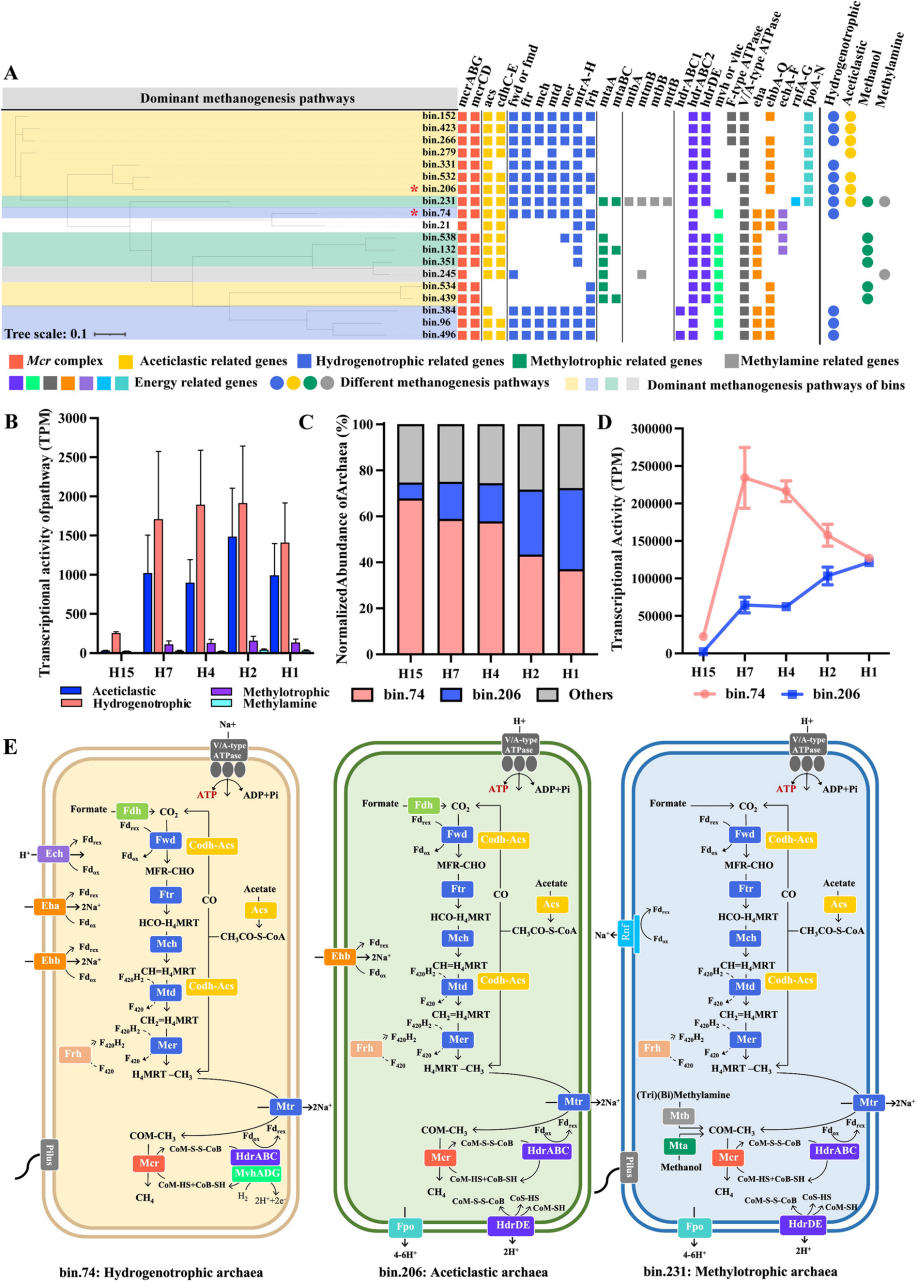
Transcriptional expression and reconstructions of the methanogenic pathways in the archaeal communities and MAGs. **A** Methanogenesis pathway reconstruction of the archaeal MAGs. **B** The change of transcriptional activities of four methanogenesis pathway with the increased organic loadings. **C** The change of relative abundance (normalized to the archaeal community) in *Methanoregulaceae* bin.74 and *Methanothrix* bin.206. **D** The change of transcriptional activities of *Methanoregulaceae* bin.74 and *Methanothrix* bin.206. **E** Methanogenesis pathway reconstruction of three representative archaeal MAGs. H15, H7, H4, H2, and H1 means the hydraulic retention times of 15, 7, 4, 2, and 1 day, respectively, and the organic loadings increased with the shortened hydraulic retention times

Archaeal methane metabolism as the terminal step in the anaerobic metabolic network plays a critical role in anaerobic carbon transformation and the natural methane flux. Methanogenesis pathway reconstruction revealed that 6 archaeal MAGs encode complete genes for both hydrogenotrophic and aceticlastic methanogenesis within the *Methanotrichales* order (Fig. 6A), and *Methanomethylovorans* sp. bin.231 (contig=1, 99.2% completeness) possessed all the genes of three methanogenesis pathways. Yet, this lineage bin.231 with a broad substrate spectrum was not active CO_2_ and acetate utilizer (only 0.35% activity in the archaeal community, Supplementary Fig. 4), reinforcing the necessity for metatranscriptomics in elucidating the ecological roles of uncultured species beyond mere pathway presence.

Hydrogenotrophic (82.0~51.6%) and aceticlastic (9.8~43.2%) methanogenesis were the prevailing forms of methane production (Fig. 6B, Supplementary Data 13). Regarding hydrogenotrophic methanogenesis, *Methanoregulaceae* bin.74 (contig=29, 99.7% completeness) overwhelmed any of other methanogens regardless of loading rates, exhibiting hydrogenotrophic activity ranging from 95.6 to 74.5% (Supplementary Fig. 7). Concerning the aceticlastic methanogenesis, three species from genus *Methanothrix*, i.e., *Methanothrix* sp. bin.206 (contig=36, 99.4% completeness), bin.532 (circular genome, 98.0% completeness), and *Methanothrix* sp. bin.266 (contig=26, 100.0% completeness), contributed to over 92% aceticlastic transcriptional activity (Supplementary Fig. 7). As above, *Methanoregulaceae* bin.74 and *Methanothrix* sp. bin.206 were recognized as the most active methane producers, given their high combined abundance (over 70%) and transcriptional activity (up to 80%) within the archaeal community (Fig. 6C, Supplementary Fig. 4). An obvious transition in overall transcriptional activity from *Methanomicrobiales* bin.74 to *Methanothrix* sp. bin.206 (Fig. 6D) highlighted the increased role of *Methanothrix* sp. bin.206 in CH_4_ production with the increase organic loading, likely due to the rising acetate amount in fresh leachate substrate. This result reinforced the environmental determinants in shaping archaeal niche dominance.

## Discussion

Studying the metabolic activity and trophic interactions among microbial communities during various stages of carbon mineralization offers insights into their contributions to the global carbon cycle, spanning implications from biotechnological management to efforts in addressing climate change. Through metatranscriptomics-guided genome-scale metabolic reconstruction, this study offered a genome-wide insight into carbon mineralization flow within the methanogenic community in details, revealing metabolic functions from the whole anaerobic community to the specific active populations, including some novel uncultured syntrophic fatty acid-oxidizing bacteria (i.e., new *Smithellaceae* and *Syntrophales* lineages) and bacteria with previously undescribed functions (e.g., sugar- and AAs-scavengers within family *VadinHA17* and *Bacteroidales*). By harnessing the long reads advantage, we recovered eight circular genomes, including two previously recognized but highly incomplete SPOB, yet-uncharacterized fermenters with versatile metabolism (*Petrimonas* sp.bin.267, etc.) and a genome from yet-to-be underexplored phylum (*Caldisericota* bin.236), advancing the holistic comprehension of anaerobic keystone species from the whole genomic perspective. In this study, we employed a 100% pathway completeness threshold (except 90% for MMC pathway) to mitigate inaccurate functional assignments. Our approach involves assessing the complete pathway transcriptional activity to pinpoint pivotal contributors in the targeted functional guild. The utilization of complete pathway transcriptional activity could address the deficiency of the *mcr*-biomarker comparison method in differentiating between hydrogenotrophic and aceticlastic methanogenesis. Furthermore, our finding emphasized the significance of metatranscriptomics in identifying the ecological functions and in situ metabolism of uncultured species. Representative example is *Syntrophobacteraceae* bin.487, it encodes the reversed Wood–Ljungdahl pathway, MMC pathway and beta-oxidation for acetate, propionate, and butyrate degradation, yet our study indicates significant transcriptional activity primarily in the MMC pathway within bin.487, consistent with the prior research classifying it as a SPOB based on isolated pure culture experiments [60]. Overall, our study establishes a framework for elucidating carbon transformations in anaerobic microbiota, linking genomic pathways of uncultured microbes with their ecological functions, and evaluating the significance of certain microbes within functional guilds via comparisons of targeted pathway activities. This framework could undoubtedly be applied to uncover novel microbes and trophic interactions in other engineered and natural ecosystems, offering significant insights into biotechnologies and global biogeochemistry.

In the phylogenetically and functionally diverse methanogenic community, the metabolic repertoire demonstrates a high level of functional redundancy across various phyla [65]. For example, *Bacteroidota*-related bacteria (bin.267, bin.202, and bin.334), along with *Thermotogota*-affiliated species (bin.190 and bin.512) and members from *Synergistota* (e.g., *Thermovirgaceae* bin.148) showed metabolic flexibility in scavenging varied AAs and sugars to generate metabolites (such as glucose, *β*-D-fructose-6P, and pyruvate) for downstream central carbon metabolism. Such a high degree of functional redundancy contributes to greater system stability, particularly in fluctuating environments with high microbial density and significant turnover of dead biomass, e.g., in anaerobic digesters with rapidly increased organic loadings. Despite having comparable metabolic capacities, different taxonomic lineages utilized functional adaptation strategies, i.e., distinct transport mechanisms and substrate preference, to mitigate fierce competition. *Thermotogota* member bin.190 transcriptionally expressed genes encoding ABC-type transporters to catalyze simple (K02056-K02058) and multiple sugar transport (K02025-K02027) as well as AA uptake, differentiating them from *Bacteroidota*-affiliated degrader bin.267 that utilizes the unique *TonB/SusC* system to facilitate sugars and AAs across the outer membrane (Supplementary Data 10). And substrate-preference-driven interactions were also observed among the fermenters from different families within *Bacteroidota* phylum, as revealed by expression comparisons of overall pathway activities. That is, *Dysgonomonadaceae* bin.267 and *VadinHA17* bin.202 favored glucose cleavage by the complete EMP pathway, whereas *Bacteroidales* bin.334 (f__4484-276) exhibited a preference for downstream pyruvate fermentation. These findings advanced the understanding how functionally diverse anaerobic populations evade substrate competition and create niche specialization, thereby forming a cohesive metabolic network within the intricate methanogenic communities.

In addition, our findings highlight that the active anaerobic lineages inhabiting nutrient-rich bioreactors exhibit remarkable metabolic flexibility and plasticity, representing an additional metabolic trait that contributes to the stability of the methanogenic community. Strong evidence of such adaptability was observed in predominant syntrophic bacterial genomes, *Smithellaceae* bin.332 and *Syntrophales* bin.292, both functioning in LCFA and propionate/butyrate metabolism. This metabolic flexibility allowed them to switch their metabolic processes responding to changes in fatty acids with different lengths and/or saturation levels in substrate. Similar to how functional redundancy across diverse phyla enhances the community-level resilience to environmental disturbances, the metabolic flexibility of anaerobic populations could provide individual lineage an adaptive survival strategy to cope with variable environmental conditions, which are also witnessed in other ecosystems, like the seafloor microbiomes [65, 66] and the gut microbiota [67, 68].

A crucial question revolves around the genomic features within each redundant functional guild that contribute to the prominence of dominant species. Integrating genomic feature analysis and transcriptional evidence of key enzymes, we conducted comparative analyses on active lineages within the same functional guilds, revealing the potential genomic determinants driving their substrate competition advantage and niche dominance. For the sugar- and AA-scavengers bin.267 and bin.202 within phylum *Bacteroidota*, their distinction from competitors could be attributed to rapid cross-membrane transport, supported by the presence and high transcriptional expression of more *susC/susD* homolog genes for protein and carbohydrate hydrolysate uptake and transport (Supplementary Fig. 8). And *Smithellaceae* bin.332 could stand out from LCFA-degrading function, primarily benefiting from exceptional bacterial motility (pilus and flagellar assembly), chemotaxis, rapid substrate-activating mechanism, and adaptive cellular degradation (Supplementary Fig. 9). The breakdown of LCFA is assumed to be adsorbed and attached by functional taxa [54, 69]. In comparison to other active LCFA-degraders, *Smithellaceae* bin.332 exhibited higher transcriptional expression of chemotaxis genes (*wspABCDEF*), type IV pilus assembly (*pilABCMNOPQTVWX*), and flagellar assembly (*flhG* and *flgP*), enhancing its mobility and attachment to LCFA molecules. Bin.332 also harbors diverse homologous for each step of beta-oxidation pathway, e.g., 15 genes for long-chain acyl-CoA synthetase (*ACSL*), allowing this syntrophic bacterium adaptive to varied types of fatty acid and/or fluctuating fatty acid concentrations in bioreactors with increased organic loadings due to differing kinetics and/or affinities of the homologous genes [6, 70]. Regarding methanogen, *Methanomicrobiales* bin.74 possessed 24 genes encoding V/A-type ATPase and four sets of membrane-bound hydrogenases (*Ech*, *Eha*, *Ehb*, and *Mbh*), a notably higher count compared to those in the bin.206 genome (9 ATPase and one hydrogenase) (Supplementary Data 12). The three clusters of alternative ATPase may confer adaptive advantages for varying growth rates or distinct concentrations of Na^+^/H^+^ in eutrophic habitats, e.g., bioreactors [70]. These specific energy-related features of bin.74 contribute to its dominance at archaeal community under low organic loading to some extent, but the extreme amount of acetate in fresh leachate substrate at high organic loading leads to a rapid increase in the activity of bin.206 that exhibits a preference for acetate utilization. Incorporating the above discoveries, the specific genetic patterns and environmental determinants foster niche specialization and ecological dominance among anaerobic community, enhancing our understanding of environmental adaptation of bacterial and archaeal lineages.

### Supplementary Information

Supplementary Material 1. 

Supplementary Material 2. 

Supplementary Material 3. 

Supplementary Material 4. 

Supplementary Material 5. 

Supplementary Material 6. 

Supplementary Material 7. 

Supplementary Material 8. 

Supplementary Material 9. 

Supplementary Material 10. 

Supplementary Material 11. 

Supplementary Material 12. 

Supplementary Material 13. 

Supplementary Material 14. 

## Data Availability

All the raw metagenomic, metatranscriptomic and nanopore sequences datasets, as well as all MAGs recovered in the current study have been deposited into the NCBI database under the BioProject accession number PRJNA1043470
