# Supplementary material for: Metatranscriptomics-guided genome-scale metabolic reconstruction reveals the carbon flux and trophic interaction in methanogenic communities
Source: Microbiome. 2024 Jul 5;12:121. doi: 10.1186/s40168-024-01830-z (PMC11225162; doi:10.1186/s40168-024-01830-z)
Supplement: Supplementary file 2 — Supplementary Material 1. [file 40168_2024_1830_MOESM1_ESM.docx]

**Supplementary Information**

**Metatranscriptomics-guided genome-scale metabolic reconstruction reveals the** **carbon flux and** **trophic interaction in** **methanogenic communities**

Weifu Yan^1^, Dou Wang^1^, Yubo Wang^1^, Chunxiao Wang^1^, Xi Chen^1^, Lei Liu^1^, Yulin Wang^1^, Yu-You Li^2^, Yoichi Kamagata^3^, Masaru K. Nobu^4^, Tong Zhang^1,5,6,7^*

^1^Environmental Microbiome Engineering and Biotechnology Laboratory, Center for Environmental Engineering Research, Department of Civil Engineering, The University of Hong Kong, Pokfulam Road, Hong Kong

^2^Laboratory of Environmental Protection Engineering, Department of Civil and Environmental Engineering, Graduate School of Engineering, Tohoku University, 6-6-06 Aza-Aoba, Aramaki, Aoba Ward, Sendai, Miyagi 980-8579, Japan

^3^Graduate School of Agriculture, Hokkaido University, Sapporo, 060-8589, Japan

^4^Institute for Extra-Cutting-Edge Science and Technology Avant-Garde Research (X-star), Japan Agency for Marine-Earth Science and Technology (JAMSTEC), Yokosuka, 237-0061, Japan

^5^ State Key Laboratory of Marine Pollution, City University of Hong Kong, Hong Kong SAR, China

^6^ School of Public Health, The University of Hong Kong, Hong Kong SAR, China

^7^ Macau Institute for Applied Research in Medicine and Health, Macau University of Science and Technology, Macao SAR, China

*Corresponding authors. E-mail addresses: [zhangt@hku.hk](mailto:zhangt@hku.hk) (Tong Zhang).

**Supplementary Information**

**Supplementary Methods**

1. Metagenomic assembly

2. Construction of phylogenetic tree

3. Carbohydrate-active enzymes and peptidase identification

4. Extraction and comparison of full-length 16S rRNA gene sequences

5. Comparison of average nucleotide identity between genomes

6. Module construction for a specific pathway

7. Identification of propionate and butyrate syntrophic degraders

**Supplementary Notes**

1. Reason for the arrangement of targeted pathways

2. Comparison of 197 MAGs with two collections of biogas microbiome

3. Novelty of eight circular MAGs

4. Identification of glycerol pathways and active degraders

5. Identification of absence of syntrophic acetate-oxidizing bacteria

6. Identification of syntrophic fatty acid-oxidizing bacteria

6.1. Identification of two described syntrophic propionate-oxidizing bacteria

6.2. Identification of two novel syntrophic bacteria

7. Overall summary about carbon flux

**Supplementary Figures**

**Supplementary Fig. 1.** The bioinformatic workflow for the metagenomics and metatranscriptomics used in this study

**Supplementary Fig. 2.** The cumulative relative abundance and transcriptional expression of 197 species-level high-quality genomes in the methanogenic community across organic loadings

**Supplementary Fig. 3.** The change of transcriptional actives in different taxonomic level in response to different organic loadings

**Supplementary Fig. 4.** The relative abundance and transcriptional expression of the top 20 most abundant MAGs under different hydraulic retention times

**Supplementary Fig. 5.** Temporal dynamics of organics components and their removal efficiencies in the substrate, digestate and permeate

**Supplementary Fig. 6.** The relative transcriptional expression of the different family-level CAZy enzymes and phylum-level taxa in response to the different hydraulic retention times

**Supplementary Fig. 7.** The change of hydrogenotrophic and aceticlastic methanogenesis transcriptional activities in the top 5 methogens in response to the different hydraulic retention times

**Supplementary Fig. 8.** The genomic feature analysis and transcriptional activities comparison of key active populations within the phylum Bacteroidota and methanogens

**Supplementary Fig. 9.** The genomic feature analysis and transcriptional activities comparison of key active long chain fatty acids-oxidizing populations

**Supplementary Datasets**

**Supplementary Data 1.** Summary of the sequencing dataset in this study

**Supplementary Data 2.** Taxonomy and relative abundance of strain-level MAGs in metagenomics and metatranscriptomics

**Supplementary Data 3.** Detailed information for 197 high-quality species-level MAGs recovered from the anaerobic communities

**Supplementary Data 4.** Summary of functional gene families related to 90 anaerobic pathways and functional modules

**Supplementary Data 5.** Comparison of assembly quality between hybrid assembly and short-reads-only assembly

**Supplementary Data 6.** Comparison of 542 MAGs with the public database and published paper

**Supplementary Data 7.** Metabolic reconstruction of 542 MAGs and their pathway transcriptional expressions under different hydraulic retention times

**Supplementary Data 8.** Pathway transcriptional expressions in specific MAGs of key anaerobic modules under different hydraulic retention times

**Supplementary Data 9.** Summary of CAZy family profile and their transcriptional expressions within the whole community and key MAGs

**Supplementary Data 10.** Key metabolic pathways reconstruction and the corresponding gene expressions of key sugars and amino acids scavengers under different hydraulic retention times

**Supplementary Data 11.** Summary of MEROPS family profile and their transcriptional expressions within the whole community and key MAGs

**Supplementary Data 12.** Key metabolic pathways reconstruction and the corresponding gene expressions of key fatty acids-degraders and methanogens under different hydraulic retention times

**Supplementary Data 13.** Four methanogenesis pathways reconstruction and their transcriptional expressions comparison within the whole archaeal community under different hydraulic retention times

**Supplementary Methods**

**1. Metagenomic assembly**

To recover as most high-quality metagenome-assembled genomes (MAGs) as possible, four approaches with different assembled strategies were selected to obtain the high-quality scaffolds/contigs as following: (1) metaSPAdes (v3.14.1) [1] for single-assemblies generated from illuminate; (2) Megahit (v1.2.9) [2] for co-assemblies generated from illuminate; (3) Unicycler (v0.4.4) [3] for single-hybrid assembly; (4) Iterative haplotype-resolved hierarchical clustering-based hybrid assembly approach (HCBHA) [4] for co-assemblies generated from illuminate and nanopore sequencing.

For short reads-based assemblers with single- and co-assembly strategies: the preprocessed clean reads of each sludge sample were independently assembled using metaSPAdes with the applied parameters ‘--meta -k 21, 33, 55’. To tackle the problem that cannot recover some unassembled contigs due to limited sequencing depth from a single sampling point, the strategy of co-assembly of all the samples was also adopted. However, metaSPAdes failed to handle all the merged datasets of approximately 113 Gb because of high computing memory. Therefore, MEGAHIT was used to co-assemble all the short reads with the parameters ‘k list: 21,29,39,59,79,99,119,141’.

For hybrid assemblers with single- and co-assembly strategies: the sludge samples from the HRT of 1, 2, 4, and 7 days were sequenced by Illumina and nanopore in pairs, and then the paired data of short reads and long reads from the same sample were used to be individually hybrid assembled by Unicycler with default parameters. Additionally, the HCBHA approach with dependencies metaFlye [5], Unicycler [3], Minimap2 [6], Bowtie2 [7], seqtk (https://github.com/lh3/seqtk) and SeqKit [8], was utilized to co-assemble all the sequencing data from short and long reads. This involved a seven-step workflow which included: 1) preparing short and long reads; 2) performing long-read-only de novo assembly using Flye with parameters ‘--nano-raw --meta -g 5m’; 3) initial binning through MetaWRAP (v1.3.2) [9] with the binning and bin_refinement modules; 4) polishing the initial bins using minimap2 and Unicycler to improve contig accuracy; 5) re-binning to further improve bin accuracy; 6) re-assembly to enhance contig contiguity; and 7) final binning to obtain highly accurate MAGs.

**2. Construction of phylogenetic tree**

A phylogenetic tree of the bacterial genomes at the strain and species level was constructed by using GTDB-Tk (v2.2.6) [10] based on 120 single-copy bacterial-specific marker genes. The identification and alignment of the marker genes were implemented using the ‘identify’ and ‘align’ modules of GTDB-Tk, respectively. A maximum likelihood tree was then accompanied using FastTree (v2.1.10) [11] with the default protein model based on the concatenated alignment of the identified markers genes, which was visualized by Interactive Tree Of Life [12].

**3. Carbohydrate-active enzymes and peptidase identification**

Genes encoding for peptidases and carbohydrate-active enzymes (CAZy) in each MAG were searched by BLASTP against publicly available peptidase (MEROPS, release 12.4) [13] and CAZy databases (version 10) [14], respectively. The analysis was performed with stringent thresholds of E-value ≤ 10 ^-20^ for peptidases, and E-value ≤ 10 ^-15^ and coverage ≥ 0.35 for CAZYs. The cellular localization of identified proteins was predicted by detecting extracellular transport signals using POSRTb (version 3.0.3) [15]. Genes associated with peptidases (or CAZy) were subsequently categorized into different families, and the expression levels of the targeted enzyme families were calculated by summing all the genes within each respective family (Supplementary Tables 6 and 7).

**4. Extraction and comparison of full-length 16S rRNA gene sequences**

Based on the annotation result of Prokka (v1.13) [16], in-house shell script was used to extract the full-length 16S rRNA gene sequences of the interesting MAGs. The extracted full-length 16S rRNA gene sequences were queried against NCBI Standard Nucleotide database for 16S ribosomal RNA sequences (Bacteria and Archaea) using Megablast, which was optimized for highly similar sequences, to search for the closest relatives (Supplementary Table 10).

**5.** **Comparison of** **average nucleotide identity between genomes**

To compare all the anaerobic species from this study with the two large collections of biogas microbiome: one collections of 2,426 draft MAGs from 56 full-scale biogas plants in China was downloaded from the GigaScience GigaDB repository (<http://gigadb.org/dataset/100842>) [17], and another collection of 1,401 assembled genomes with medium- and high-quality from various biogas reactors was obtained the NCBI database under the bioproject PRJNA602310 [18].

For evaluating the novelty of the active functional genomes in different anaerobic guilds, the literature relevant to the functional active species was obtained following a taxonomy-based approach. Subsequently, the corresponding genome file was obtained according to the GenBank assembly accession for comparison analysis. For the syntrophic propionate-oxidizing bacteria: The genomes files of Ca. Propionivorax syntrophicum F70 (GenBank assembly accession: GCA_900750585.1) was downloaded from European Nucleotide Archive under the project ID PRJEB31310 [19]. For the amino acids scavengers in the Bacteroidetes: Five genomes files of uncultured Bacteroidales populations (GenBank assembly accession: GCA_009881065.1, GCA_009877225.1, GCA_009877185.1, GCA_009877155.1 and GCA_009877105.1) was downloaded from NCBI GenBank under the accession WXFB00000000 [20].

The comparison between genomes were then performed using fastANI (version 1.33) [21] by computing whole-genome average nucleotide identity (ANI) between genomes, whose results are provided in Supplementary Data S6.

**6. Module construction for a specific pathway**

Due to the lack of modules for some arranged pathways, the modules were constructed based on rules referred to the KEGG existing modules. The rules for constructing modules for the arranged pathways mainly follow the criteria: (1) the number of steps in a specific pathway indicated by blank spacing; (2) the number of enzymes catalyzing the same reaction in a step were included in the same parenthesis ‘()’; (3) the number of subunits of an enzyme complex is indicated by the symbol ‘+’.

**7.** **Identification of** **propionate and butyrate syntrophic degraders**

Due to the high similarity between genes and pathways involved in propionate/butyrate catabolism and propionate/butyrate fermentation, the identified syntrophic propionate and butyrate oxidizers was considered valid if the target genome contained a (nearly) complete pathway for propionate or butyrate oxidation (methylmalonyl-CoA pathway or butyrate beta-oxidation pathway) and electron transfer reactions that were compatible with the re-oxidation of electron carriers involved [22]. Specifically, syntrophic propionate and butyrate oxidizers must also exhibit transcriptional activities in the genes related to (1) interspecies electron transfer, such as hydrogenases and formate dehydrogenases [19]; (2) four types of flavin-based electron bifurcation/confurcation, i.e., electron-transferring flavoprotein (EtfAB)-containing complexes, NAD (P)H dehydrogenase (NuoF homologue) complexes, heterodisulfide reductase (HdrABC)- containing complexes, and NADH-dependent ferredoxin-NADP reductase (NfnAB) [23]; (3) reverse electron transport mechanisms, e.g., iron-sulfur-binding reductases and the Fix system for butyrate degradation [24,25]. The detailed metabolic reconstruction about the key propionate and butyrate oxidizers in this study are provided in Supplementary Data S12.

**Supplementary Notes**

**1. Reason for** **the arrangement of targeted pathways**

The selection of specific pathways is primarily driven by the composition and quantities of compounds existing in the influent fresh leachate, which serve as substrates for anaerobic microorganisms. The comprehensive information about the substrate properties was also provided in our prior study [26].

The complex polymers in the fresh leachate: The protein measurements results showed that the average concentrations of total and dissolved proteins were 1895 ± 549 mg/L and 790 ± 196 mg/L, respectively. And the average concentrations of total and dissolved carbohydrates exhibited similar levels to those of proteins, standing at 1246 ± 569 mg/L and 624 ± 332 mg/L, respectively. In addition, the amount of lipids has not been determined in the fresh leachate, however, it could be speculated that there might be certain amounts of fats and/or greases present in the substrate given that Hong Kong does not have a garbage sorting policy, which also indicated by the macroscopic grease floating on the surface of the leachate.

The volatile fatty acids in the fresh leachate: A total of 11 volatile fatty acids (VFAs) were detected, with acetate, propionate, butyrate, valeric acid, and hexanoic acid being the predominant VFAs. Significantly, the influent displayed notably high concentrations of acetate, propionate, and butyrate, reaching levels of up to 7,359 mg/L, 3,536 mg/L, and 6,347 mg/L, respectively. Given the absence of VFA accumulation during the 215-day operation, it can be deduced that a syntrophic relationship exists between syntrophic bacteria and methanogenic archaea in this anaerobic ecosystem.

**2. Comparison of 197 MAGs with two collections of biogas microbiome**

To evaluate the novelty and quality improvement of assembled genomes in our study, we performed a species-level comparison of 197 high-completeness MAGs to two large sets of anaerobic MAGs. The first set comprised 2,426 MAGs with ≥80% completeness and ≤10% contamination from 56 full-scale biogas plants in China [17], while the second set consisted of 1,401 MAGs (completeness ≥ 70% and contamination < 10%) derived from 134 public metagenomes from various biogas reactors [18]. The comparison result pointed out that only 68 MAGs with ≥90% completeness were clustered to the two public biogas microbiome datasets at the 95% ANI level (Supplementary Data S6). This could probably be attributed to the distinct substrate components used in various studies. Lab-scale anaerobic reactors and full-scale biogas plants in China predominantly utilized manure (e.g., pig and cow manure) and sludge, while in contrast, our study focuses on realistic fresh leachate as a substrate, which possesses different components compared to manure and sludge. These finding highlights both the remarkable uniqueness of anaerobic MAGs in our study and emphasizes the vast phylogenetic and metabolic traits of unexplored anaerobic lineages in diverse intricate ecosystems. Additionally, according to standards of minimum information about a metagenome-assembled genome (MIMAG) [27], only 4 MAGs from our study had lower quality than those in the public datasets, indicating a substantial improvement in the quality of 94.11% of the 68 MAGs in the previous public dataset. Such high proportion of retrieval of novel high-quality anaerobic lineages in our study suggested that long-reads-based metagenomics could be regarded as a powerful approach to discover novel uncultured microbial populations with high qualities, thereby increasing the accuracy for functional analysis in anaerobic microbiome.

**3. Novelty of eight circular MAGs**

One significant improvement of hybrid assemblies incorporating nanopore long reads was the successful recovery of eight circular MAGs (cMAG), including one archaeal and seven bacterial genomes. The average completeness and contamination of these cMAG were 97.62% and 1.47%, respectively (Supplementary DataS3).

The 8 cMAGs were compared with 2,426 MAGs recovered from 56 full-scale biogas plants in China [17] and 1,401 MAGs derived from various biogas reactors [18], as well as the Genome Taxonomy Database (release207_v2) to estimate the novelty of these cMAGs. Three homologous species (MCAAHP04F1.bin.169, MCHBJC03F1.bin.125 and GCA_012838585.1_ASM1283858v1_genomic) were identified in the biogas MAGs dataset, sharing ANI of 96.49%, 99.51% and 98.68% with bin.532, bin.218 and bin.486, respectively. In GTDB database (R07-RS207), five species (GCA_002305915.1, contigs=124; GCA_002069095.1, contigs=299; GCA_012838585.1, contigs=278; GCA_013314815.1, contigs=245; GCF_016706325.1, contigs=3) shared high ANI of 99.26%, 98.67%, 98.88%, 98.04% and 98.73% with bin.218, bin.267, bin.486, bin.487 and bin.532, respectively (Supplementary DataS3). These five homologous genomes in public databases were mostly assembled by short-reads-based metagenomic data with an average of 190 contigs, suggesting that the cMAGs in this study represent the first circularized, nearly complete genomes for their corresponding species. In addition, the remaining 3 cMAGs showed previously unknown and novel genomic features, for they shared low ANI (all ANI below 82.48%) with species in the databases.

In addition, the full-length 16S rRNA genes were extracted from these cMAG, which were further used to search against the NCBI 16S rRNA sequence database. The comparison results (Supplementary DataS3) showed that only two cMAGs, bin.532 and bin.487, had high identities with *Methanothrix soehngenii* GP6 (99.86% identity) and *Syntrophobacterium sulfatireducens* strain TB8106 (99.03% identity), respectively. The remaining 6 genomes exhibited quietly low identities with their corresponding top hits, with the highest and lowest identity of 94.83 and 82.36%, respectively. According to the 16S rRNA-based taxonomy standard [28], bin.267, bin.218, bin.276 and bin.486 could be identified as new microbes affiliated with a new genus (16S rRNA identity of <95%), while bin.314 and bin.236 might be the representatives of potential new families (16S rRNA identity of <87%).

Combined the results of ANI comparison between genomes and 16S rRNA-based classification, it could be concluded that three complete genomes in our study were proposed as potential a new genus (*Dehalobacteriia* sp. bin.276), a new family (*Anaerolineae* bin.314) and a new order (*Caldisericia* bin.236). This result suggests that the hybrid assembly incorporating long reads not only improved the completeness and continuity of assembled genomes but also unravel the previously hidden genomic and taxonomic information by recovering the microbial dark matters.

**4. Identification of glycerol** **pathways and active degraders**

Glycerol, as another of the lipolysis products of lipids, undergoes further degradation through either one reductive pathway for 1,3-propanediol formation, or two alternative oxidative pathways to the generate glycerone phosphate [29]. The metabolites then flow into the central carbon metabolism. Of note, the complete genes of the reductive pathway were not detected in any of the genomes, and only a small number (22) of genomes with minimal transcriptional expressions have the genetic potential for the glycerol oxidative pathway where glycerol is converted to dihydroxyacetone (Supplementary DataS8). These results indicated that they are not the primary degradation routes for glycerol in this methanogenic system. By contrast, a significant proportion (51.84%) of the genomes (Fig. 4) encodes the necessary genes for alternative oxidative pathway involving the conversion of glycerol to sn-glycerol 3-phosphate, indicating that they might degrade glycerol by a membrane-bound glycerol-3-phosphate dehydrogenase (*glpABC*). Further examination of the transcriptional expression reveals that species such as bin.189 and bin.307 which belong to *Chloroflexota* phylum, as well as *Bacteroidota*-affiliated bin.202 and *Mestoga* sp. bin.190 were actively glycerol degraders. Consistent with this function, these populations also transcriptionally expressed genes encoding glycerol-related transporters, i.e., glycerol uptake facilitator protein (*glpF*) in bin.189 and sn-glycerol 3-phosphate transport system (*ugpABE*) in bin.190.

**5. Identification of** **absence of syntrophic acetate-****oxidizing bacteria**

Methanogenesis from acetate involves two processes: aceticlastic methanogenesis and/or bacterial acetate oxidation via the reversed Wood–Ljungdahl pathway [30,31]. After the reconstruction of the reversed Wood–Ljungdahl pathway, only five genomes were discovered to contain the required genes for this pathway. This limited number could be attributed to the stringent criteria used for pathway filtering, which required 100% completeness for all nine steps of the reversed Wood–Ljungdahl pathway. Surprisingly, none of the genomes exhibited high transcriptional expression of the reversed Wood–Ljungdahl pathway, even when the pathway filter's cutoff was lowered, indicating that bacterial acetate oxidation via the reversed Wood–Ljungdahl pathway is not the major conversion metabolism of acetate under this anaerobic condition.

In this study, *Mesotoga* sp.bin.190 exhibited a remarkably high level of transcriptional activity, contributing to 20.3% of the mapped transcriptomes at HRT=15 days. And previous investigations reported that *Mesotoga* population (bin.190) could metabolize acetate by alternative glycine cleavage system and tetrahydrofolate pathway rather than the reversed Wood–Ljungdahl pathway [32]. However, the transcriptional expression of this pathway comprises a few fractions (approximately 5.1%) of the overall metatranscriptomic activity observed in *Mesotoga* sp. bin.190, suggesting that acetate oxidation is not the primary metabolism of *Mesotoga* population in this methanogenic environment. Furthermore, the low activity of *Mesotoga* (4.25% of the mapped metatranscriptome) is not comparable to aceticlastic *Methanothrix* (15.76% of the mapped metatranscriptome) when facing extremely high acetate concentrations at higher OLR. This result further supports that *Mesotoga* have few contributions to acetate oxidation, probably due to the unfavorable energetics it faces under standard thermodynamics resulting in its inability to compete with aceticlastic archaea (bin.206). Overall, acetate oxidation in this methanogenic reactor is primarily driven by aceticlastic methanogens rather than bacterial metabolic pathways.

**6. Identification of syntrophic fatty acid-oxidizing bacteria**

**6.1. Identification of two described syntrophic propionate-oxidizing bacteria**

*Syntrophobacteraceae* bin.487 (circular genome, 99.5% completeness), and *Pelotomaculaceae* bin.218 (circular genome, 100% completeness) as two active participants in propionate degradation. Based on ANI comparisons and 16S rRNA alignment, bin.487 and bin.218 exhibited high similarities with known syntrophic propionate-oxidizing bacteria (SPOB) [19,33], The full-length 16S rRNA gene of bin.487 showed 99.0% similarity with the propionate-oxidizing isolate, *Syntrophobacter sulfatireducens* strains TB8106T (Supplementary DataS7) [33], while bin.218 shared a high ANI (99.6%) with *Ca.* *Propionivorax syntrophicum* F70, a recently identified SPOB commonly found in Danish digesters [19]. To date, however, the complete genomic information of *Syntrophobacterium sulfatireducens* strain TB8106 was not available in the current public database. And *Ca.* *Propionivorax syntrophicum* F70 was highly fragmented genome with completeness of 74.7% and contig of 404. By contrast, the cMAGs bin.487 and bin.218 obtained in our study provided the corresponding complete genome features, including multiple copies of rRNA operons and repeated regions (Supplementary DataS7), thereby enhancing the comprehension of their involvement in propionate metabolism on a genomic scale.

**6.2. Identification of two novel syntrophic bacteria**

The assembled genomes bin.292 (contig=13, 90.3% completeness) and bin.332 (contig=26, 92.9% completeness) were identified as the key active long chain fatty acids degraders, and they also exhibited high transcriptional expression of propionate oxidation and butyrate beta-oxidation pathway, respectively. Their phylogenetic positions were determined by conducting genome ANI comparison and 16S rRNA gene sequence alignment against the GTDB and NCBI nucleotide database.

**6.2.1. Phylogenetic position of Bin.292, a member of the family *Syntrophaceae***

Bin.292, a 2.38 Mbp draft genome (completeness 90.32%; contamination 1.94%; 13 contigs) exhibited an ANI similarity of 79.64% to *Syntrophaceae* bacterium UBA6252 (GCA_002441265.1), which is categorized as: Bacteria; Desulfobacterota; Syntrophia; Syntrophales; UBA6807; UBA6807; UBA6807 sp002441265, according to GTDB (release214) classification. Based on the annotation result from Prokka, the full-length of 16S rRNA gene (a 1566-bp long gene sequence) was extracted from the Bin.292 genome, which showed 91.38% identity with *Syntrophus aciditrophicus* strain SB (NR_102776.1), and 90.78% identity with *Syntrophus buswellii* strain DM-2 (NR_102776.1); the former is a significant example of a syntrophic butyrate-oxidizing bacterium involved in the degradation of fatty and aromatic acids [24], while the latter is a bacterium engaged in syntrophic butyrate or benzoate oxidation [34]. Based on the genome ANI comparison coupled with phylogenetic analysis indicates that bin.292 is probably a new genus within the family UBA6807 (based on GTDB classification), or *Syntrophaceae* (based on the 16S rRNA gene sequence in NCBI taxonomy).

**6.2.2. Phylogenetic position of Bin.332, a member of the family *Smithellaceae***

Bin.332, a 2.65 Mbp MAG (completeness 92.90%; contamination 3.89%; 26 contigs) displayed a quite low similarity, with an ANI below 80%, to any of the reference genomes in the GTDB (release214) database, as reflected by "N/A" in the "fastani_ani" column of the GTDB result. Due to the lack of full length 16S rRNA gene sequence within Bin.332 genome, it was not feasible to perform alignment with the NCBI 16S rRNA gene database. Hence, according to the GTDB analysis, bin. 332 is indicative of a potential new species or a higher taxonomic classification within the *Smithellaceae* family.

**7. Overall summary about carbon flux**

To unravel the anaerobic keystone guilds and in-situ metabolic activity of key species, metatranscriptomics-guided genome-scale metabolic reconstruction was used to explore metabolic functions from the overall microbial community to the specific active populations. In details, *Bacteroidales*-affiliated bacteria (bin.267, bin.202 and bin.334) and populations from *Mesotoga* sp. (bin.190 and bin.512) showed metabolic flexibility in scavenging varied AAs and sugars to generate metabolites (such as glucose, *β*-D-fructose-6P and pyruvate) for central carbon metabolism, then these metabolic intermediates were utilized by the fermentative bacteria (such as bin.218 and bin.479 from family *Pelotomaculaceae* and *VadinHA17*, respectively). In addition, species from *Chloroflexota* phylum, i.e., bin.189 and bin.307, and *Bacteroidota*-affiliated bin.202 displayed significant transcriptional expression in glycerol oxidative pathway to generate glycerone-P, which also flow into the central carbon module. Syntrophic active populations from the order *Syntrophales,* *Syntrophobacterales* and *Desulfotomaculales,* including the previously known SPOB bin.487 and several novel bacteria (bin.332, bin.292 and bin.218) functioned in LCFA and SCFA metabolism to produce acetate and formate. The hydrogenotrophic methanogens represented by *Methanoregulaceae* bin.74 and aceticlastic methanogens represented by *Methanothrix* sp. bin.206 both contributed to methane production by utilizing H_2_/CO_2_ or formate and acetate.

**Supplementary Figures**


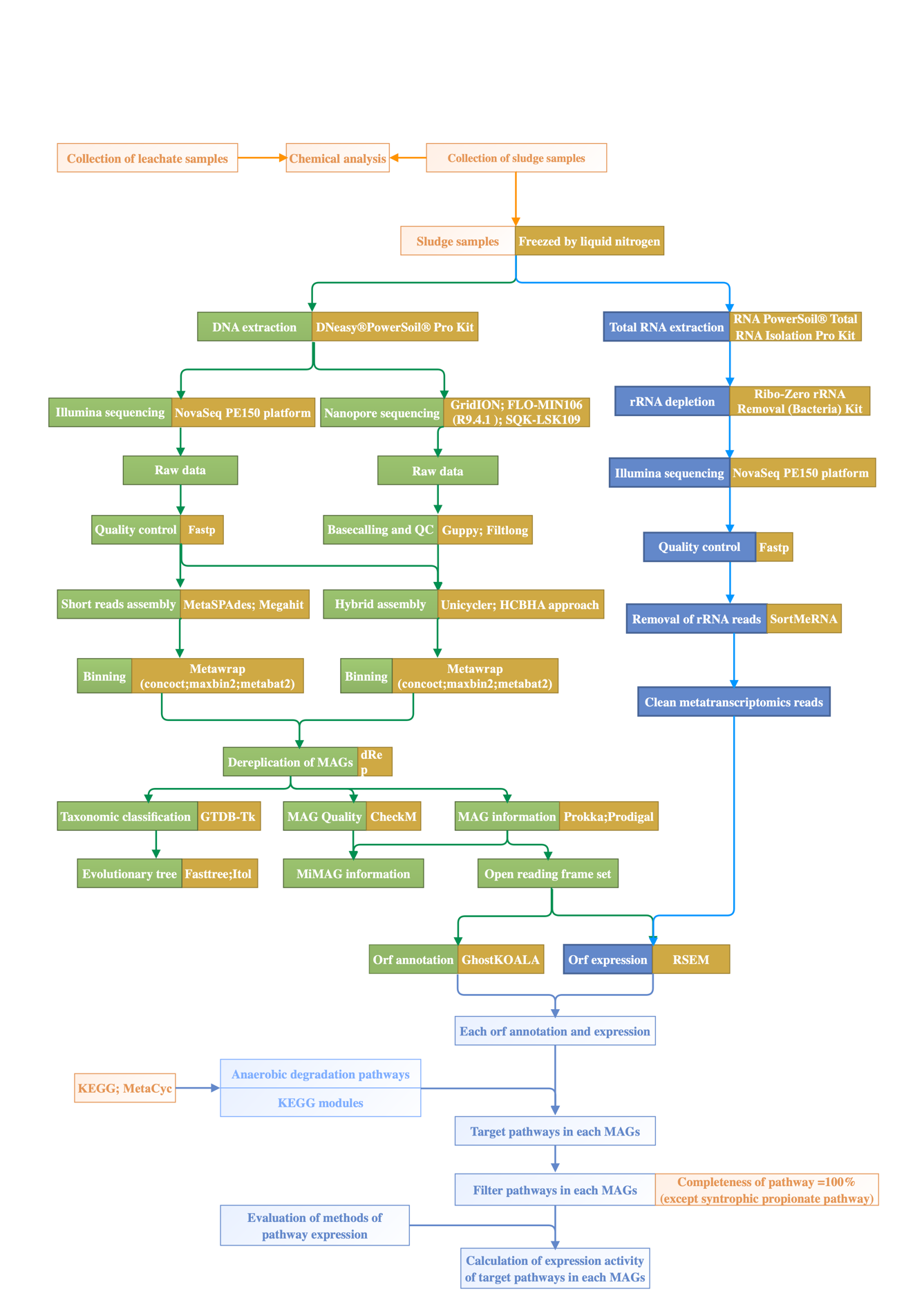


**Supplementary Fig. 1. The bioinformatic workflow for the metagenomics and metatranscriptomics used in this study.**


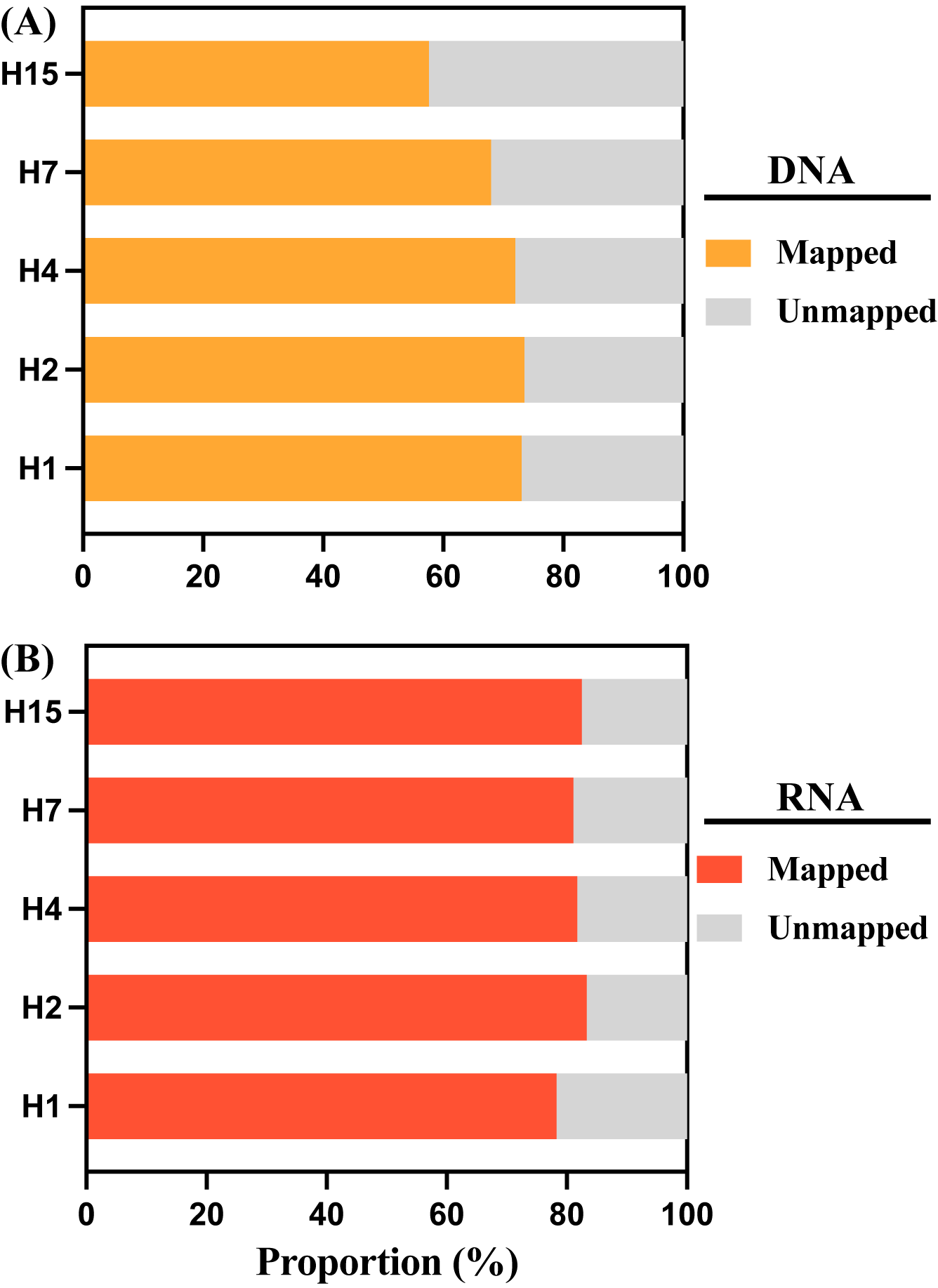


**Supplementary Fig. 2. The cumulative relative abundance and transcriptional expression of 197 species-level high-quality genomes in the methanogenic community** **across organic loadings.** (A) The summed relative abundance of 197 high-quality genomes in the methanogenic community. (B) The summed relative transcriptional expression of 197 high-quality genomes in the methanogenic community. H15, H7, H4, H2 and H1 means the hydraulic retention times of 15, 7, 4, 2 and 1 day, respectively, and the organic loadings increased with the shortened hydraulic retention times.


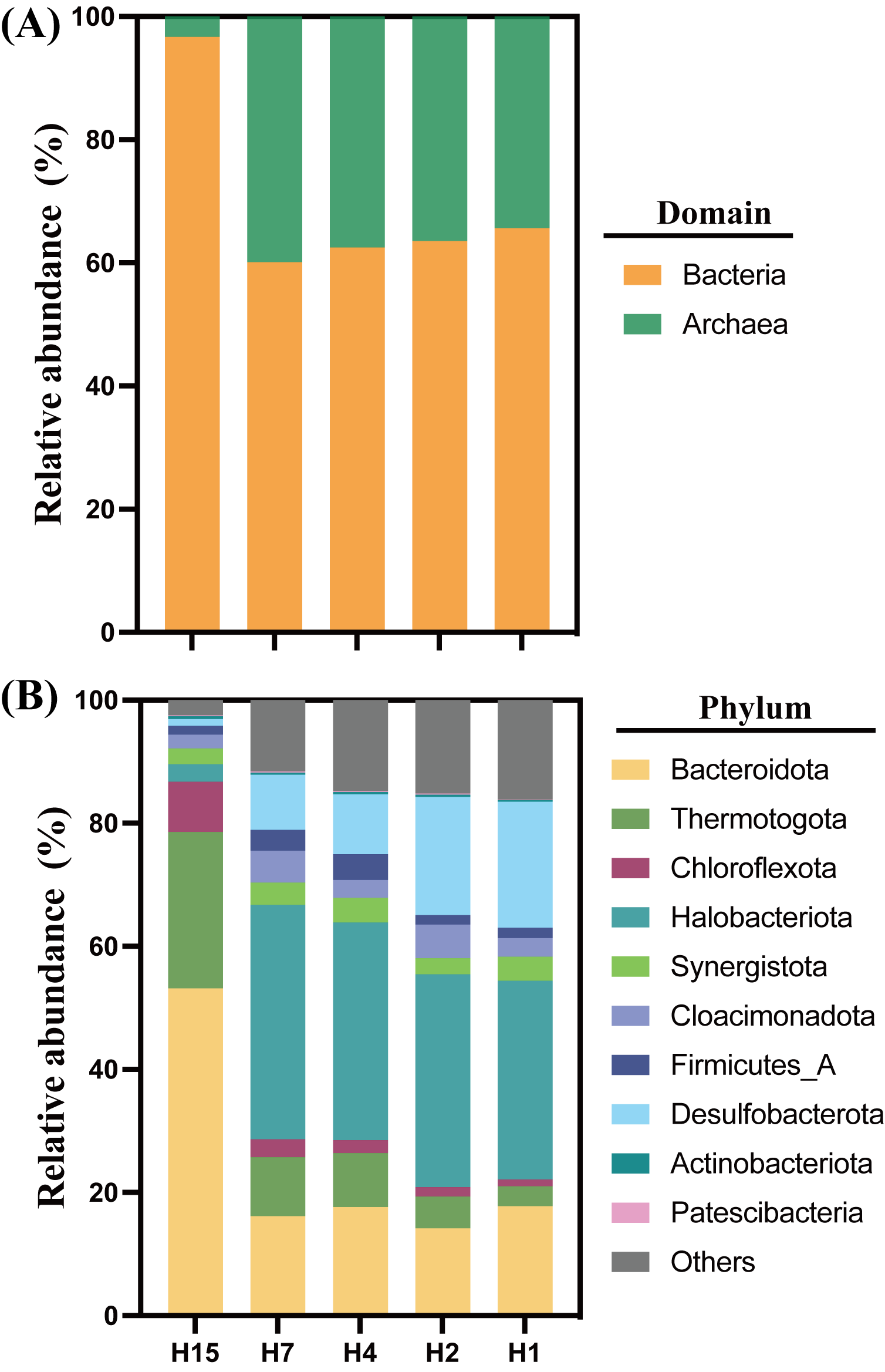


**Supplementary Fig. 3.** **The change of transcriptional actives in different taxonomic level in response to different organic loadings.** (A) The transcriptional variation of domain-level taxa across the organic loadings; (B) The transcriptional variation of phylum-level taxa across the organic loadings. H15, H7, H4, H2 and H1 means the hydraulic retention times of 15, 7, 4, 2 and 1 day, respectively, and the organic loadings increased with the shortened hydraulic retention times.


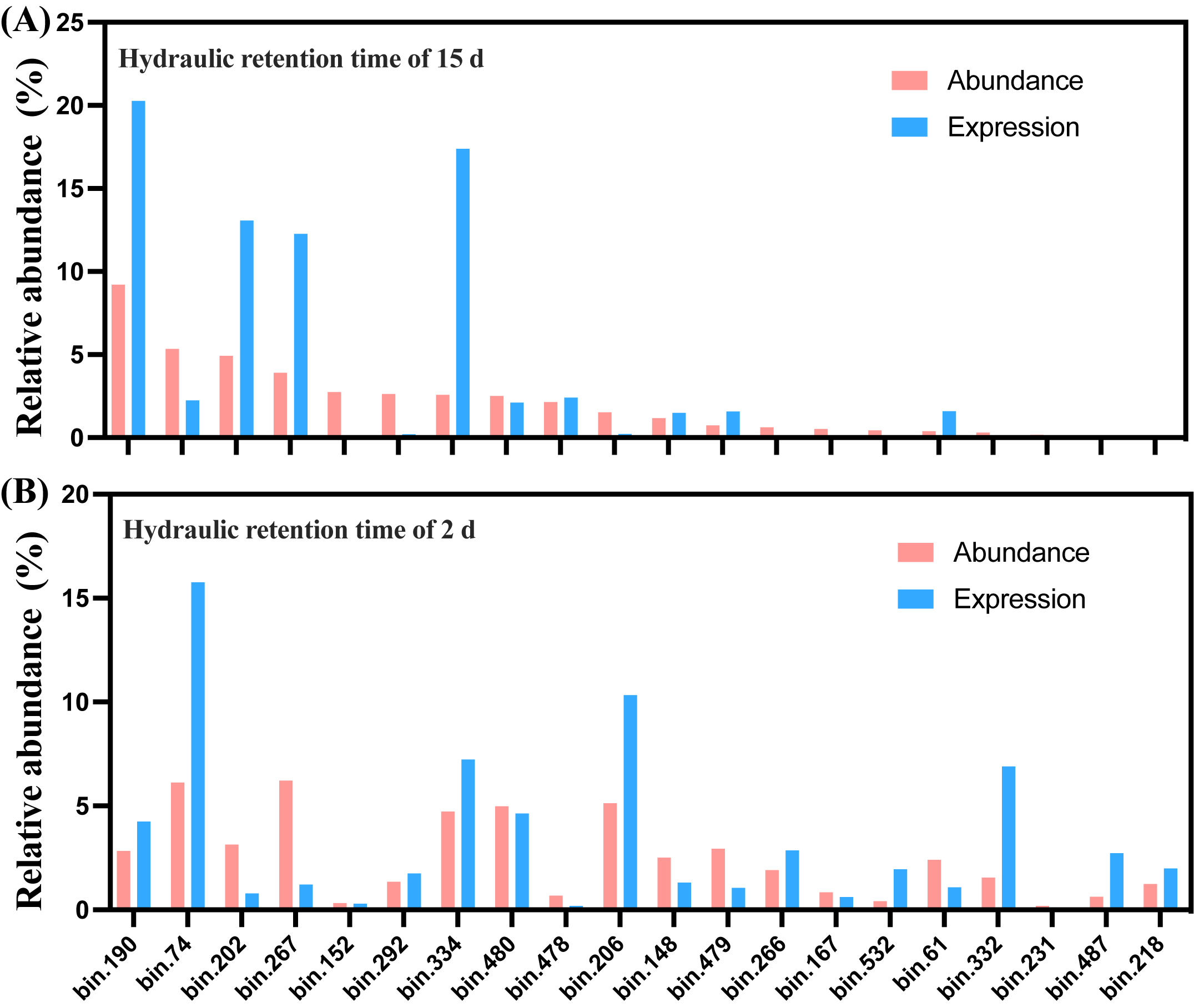


**Supplementary Fig. 4. The relative abundance and transcriptional expression of the top 20 most abundant MAGs under different hydraulic retention times.** (A) The relative abundance and transcriptional expression of the top 20 most abundant MAGs in the hydraulic retention time of 15 day; (B) The relative abundance and transcriptional expression of the top 20 most abundant MAGs in the hydraulic retention time of 2 day.


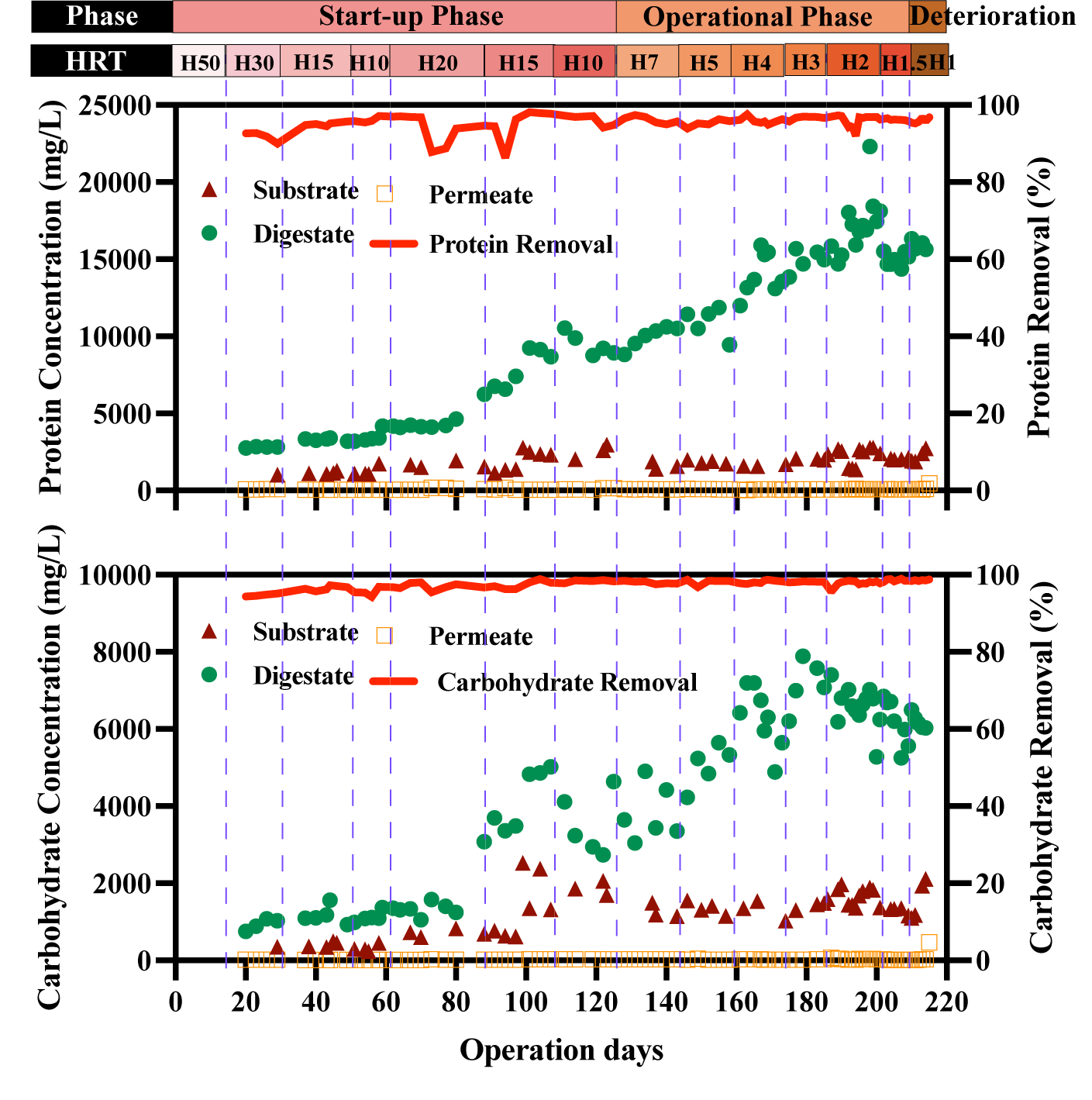


**Supplementary Fig. 5. Temporal dynamics of organics components and their removal efficiencies in the substrate, digestate and permeate.** Substrate means fresh leachate; Digestate means sludge in reactor; Permeate means effluent.


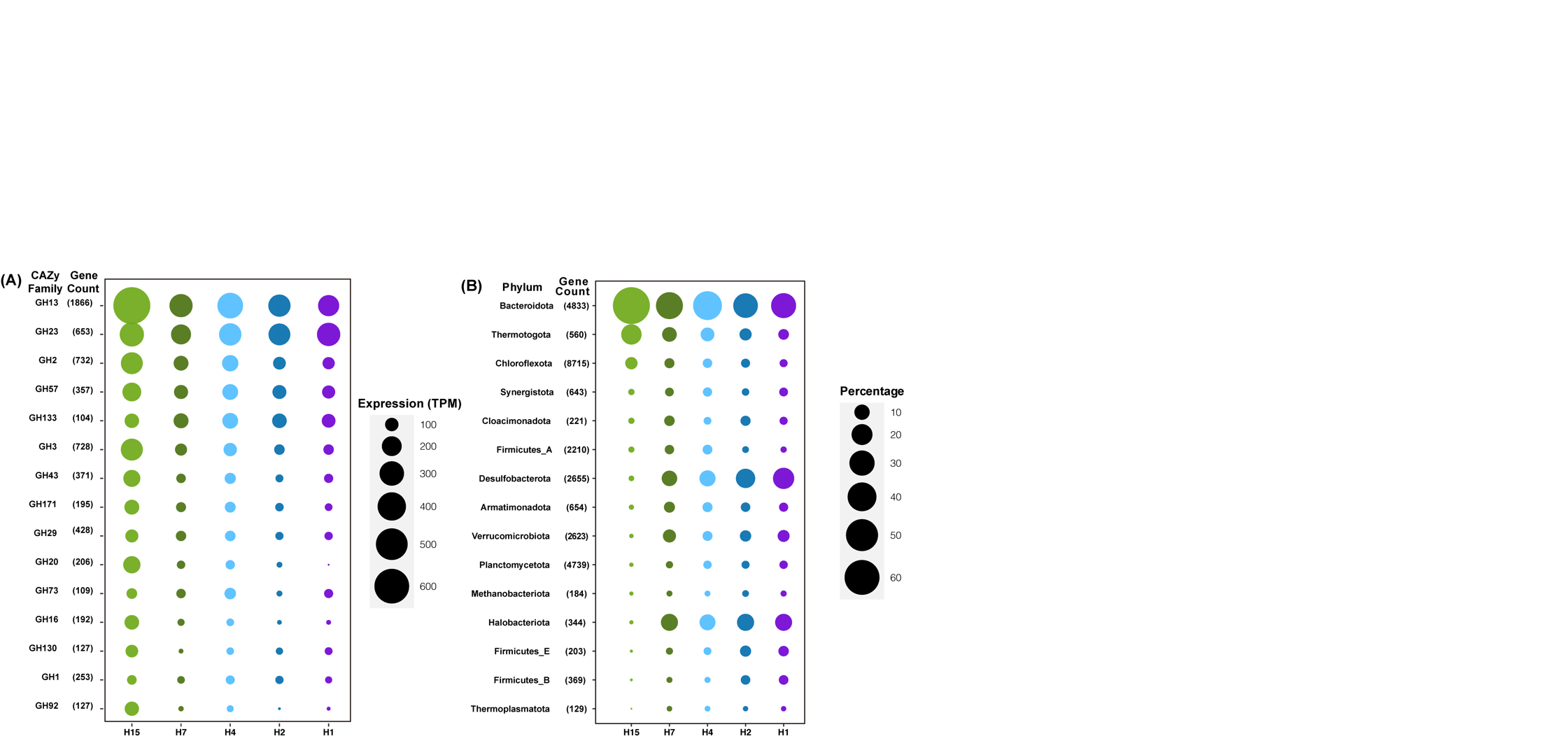


**Supplementary Fig. 6. The relative transcriptional expression of the different family-level CAZy enzymes and phylum-level taxa in response to the different hydraulic retention times.** (A) The change of transcriptional activities in different family-level CAZy enzymes responding to the increased organic loadings; (B) The change of transcriptional activities in different phylum-level taxa responding to the increased organic loadings. Gene count means the total predicted number of genes belonging to the CAZy enzymes or phylum in the whole community. H15, H7, H4, H2 and H1 means the hydraulic retention times of 15, 7, 4, 2 and 1 day, respectively, and the organic loadings increased with the shortened hydraulic retention times.

**Supplementary Fig. 7. The change of hydrogenotrophic and aceticlastic methanogenesis transcriptional activities in the top 5 methogens in response to the different hydraulic retention times.** (A) The change of hydrogenotrophic methanogenesis transcriptional activities in the top 5 methogens responding to the increased organic loadings. (B) The change of aceticlastic methanogenesis transcriptional activities in the top 5 methogens responding to the increased organic loadings. The relative transcriptional activities were normalized to the archaeal community. H15, H7, H4, H2 and H1 means the hydraulic retention times of 15, 7, 4, 2 and 1 day, respectively, and the organic loadings increased with the shortened hydraulic retention times.


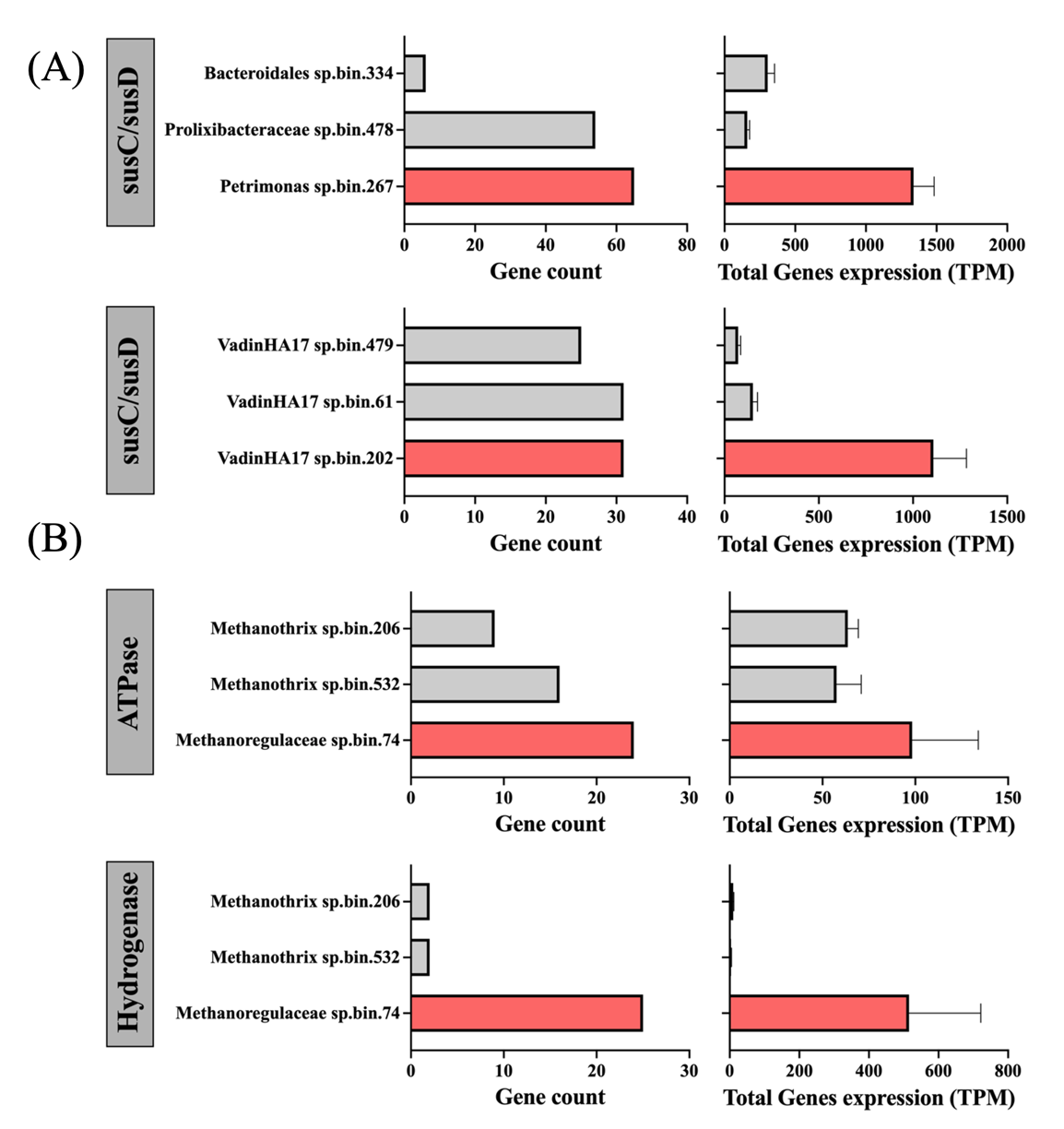


**Supplementary Fig. 8. The genomic feature analysis and transcriptional activities comparison of key active populations within the phylum Bacteroidota and methanogens.** (A) The susC/susD gene count and their transcriptional expression in the active populations within the phylum Bacteroidota in the hydraulic retention time of 15 day. (B) The ATPase and hydrogenase gene count and their transcriptional expression in the three active methanogens in the hydraulic retention time of 2 day.


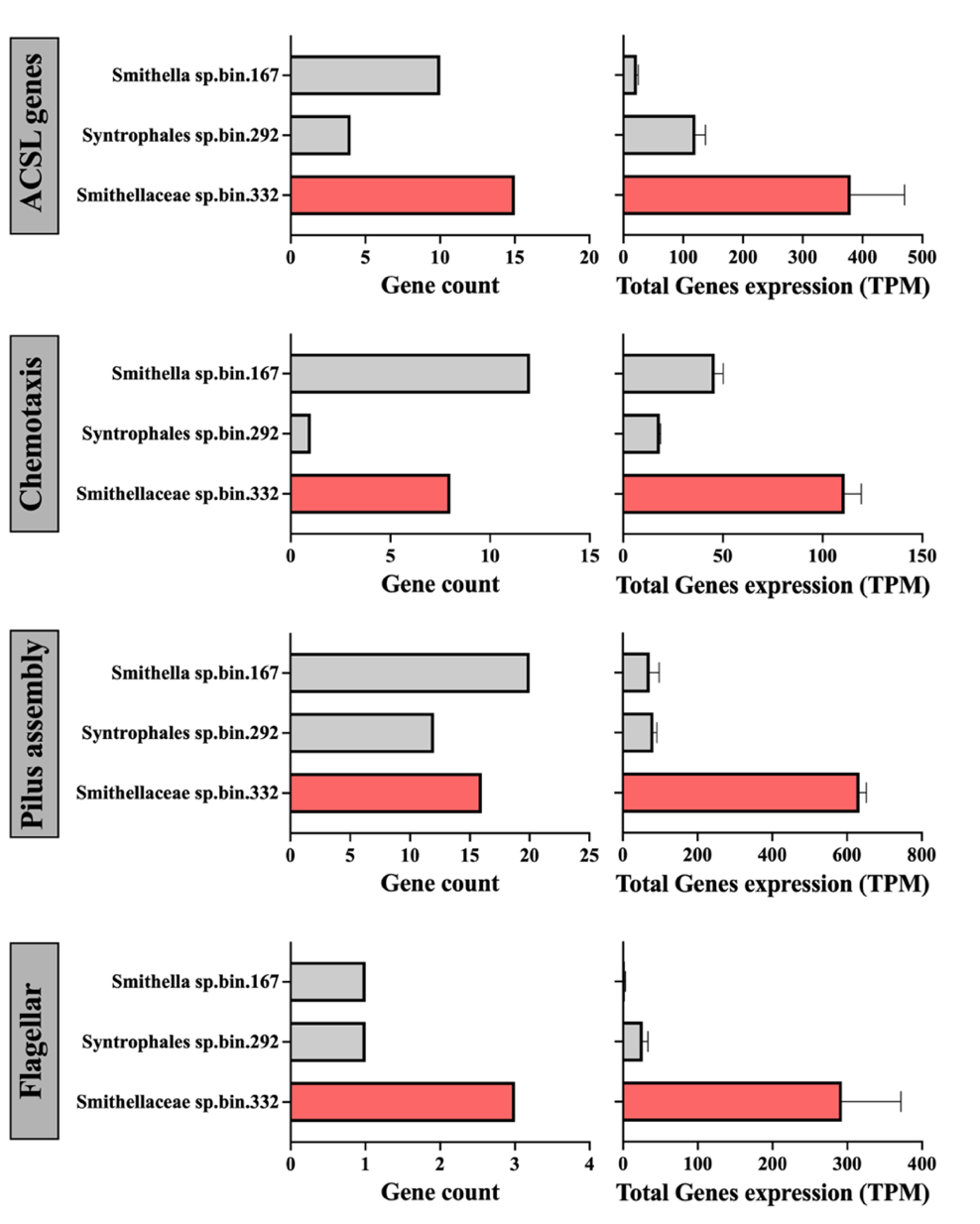


**Supplementary Fig. 9. The genomic feature analysis and transcriptional activities comparison of key active long chain fatty acid-oxidizing populations.** The gene count related to long chain fatty acids activation gene, chemotaxis, pilus assembly and flagellar, and their transcriptional expression in the three active LCFA-degrading populations in the hydraulic retention time of 2 day.

**Reference**

1. Nurk S, et al. metaSPAdes: a new versatile metagenomic assembler. Genome Res. 2017;27:824-34.

2. Li D, et al. MEGAHIT: an ultra-fast single-node solution for large and complex metagenomics assembly via succinct de Bruijn graph. Bioinformatics. 2015;31:1674-76.

3. Wick R R, et al. Unicycler: Resolving bacterial genome assemblies from short and long sequencing reads. PLOS Comput Biol. 2017;13:e1005595.

4. Liu L, et al. Charting the complexity of the activated sludge microbiome through a hybrid sequencing strategy. Microbiome. 2021;9:205.

5. Kolmogorov M, et al. metaFlye: scalable long-read metagenome assembly using repeat graphs. Nat Methods. 2020;17:1103-10.

6. Li H. Minimap2: pairwise alignment for nucleotide sequences. Bioinformatics. 2018;34:3094-100.

7. Langmead B, S L Salzberg. Fast gapped-read alignment with Bowtie 2. Nat Methods. 2012;9:357-59.

8. Shen W, et al. SeqKit: A cross-platform and ultrafast toolkit for FASTA/Q file manipulation. Plos One. 2016;11:e0163962.

9. Uritskiy G V, J DiRuggiero, J Taylor. MetaWRAP—a flexible pipeline for genome-resolved metagenomic data analysis. Microbiome. 2018;6:158.

10. Chaumeil P-A, et al. GTDB-Tk v2: memory friendly classification with the genome taxonomy database. Bioinformatics. 2022;38:5315-16.

11. Price M N, P S Dehal, A P Arkin. FastTree 2 – approximately maximum-likelihood trees for large alignments. Plos One. 2010;5:e9490.

12. Letunic I, P Bork. Interactive Tree Of Life (iTOL) v5: an online tool for phylogenetic tree display and annotation. Nucleic Acids Res. 2021;49:W293-W96.

13. Rawlings N D, et al. The MEROPS database of proteolytic enzymes, their substrates and inhibitors in 2017 and a comparison with peptidases in the PANTHER database. Nucleic Acids Res. 2017;46:D624-D32.

14. Drula E, et al. The carbohydrate-active enzyme database: functions and literature. Nucleic Acids Res. 2021;50:D571-D77.

15. Yu N Y, et al. PSORTb 3.0: improved protein subcellular localization prediction with refined localization subcategories and predictive capabilities for all prokaryotes. Bioinformatics. 2010;26:1608-15.

16. Seemann T. Prokka: rapid prokaryotic genome annotation. Bioinformatics. 2014;30:2068-69.

17. Ma S, et al. A microbial gene catalog of anaerobic digestion from full-scale biogas plants. GigaScience. 2021;10.

18. Campanaro S, et al. New insights from the biogas microbiome by comprehensive genome-resolved metagenomics of nearly 1600 species originating from multiple anaerobic digesters. Biotechnol Biofuels. 2020;13:25.

19. Hao L, et al. Novel syntrophic bacteria in full-scale anaerobic digesters revealed by genome-centric metatranscriptomics. ISME J. 2020;14:906-18.

20. Mei R, et al. Metagenomic and metatranscriptomic analyses revealed uncultured Bacteroidales populations as the dominant proteolytic amino acid degraders in anaerobic digesters. Front Microbiol. 2020;11.

21. Jain C, et al. High throughput ANI analysis of 90K prokaryotic genomes reveals clear species boundaries. Nat Commun. 2018;9:5114.

22. Nobu M K, et al. Catabolism and interactions of uncultured organisms shaped by eco-thermodynamics in methanogenic bioprocesses. Microbiome. 2020;8:111.

23. Jin Y, Y Lu. Syntrophic Propionate Oxidation: One of the Rate-Limiting Steps of Organic Matter Decomposition in Anoxic Environments. Appl Environ Microbiol. 2023;89:e00384-23.

24. McInerney M J, et al. The genome of Syntrophus aciditrophicus: Life at the thermodynamic limit of microbial growth. P Natl Acad Sci USA. 2007;104:7600-05.

25. Sieber J R, et al. The genome of Syntrophomonas wolfei: new insights into syntrophic metabolism and biohydrogen production. Environ Microbiol. 2010;12:2289-301.

26. Yan W, et al. Treatment of fresh leachate by anaerobic membrane bioreactor: On-site investigation, long-term performance and response of microbial community. Bioresour Technol. 2023;383:129243.

27. Bowers R M, et al. Minimum information about a single amplified genome (MISAG) and a metagenome-assembled genome (MIMAG) of bacteria and archaea. Nat Biotechnol. 2017;35:725-31.

28. Zhang Y, T Zhang. Culturing the uncultured microbial majority in activated sludge: A critical review. Crit Rev Env Sci Tec. 2022:1-24.

29. Elsamadony M, et al. Advances towards understanding long chain fatty acids-induced inhibition and overcoming strategies for efficient anaerobic digestion process. Water Res. 2021;190:116732.

30. Mayumi D, et al. Carbon dioxide concentration dictates alternative methanogenic pathways in oil reservoirs. Nat Commun. 2013;4:1998.

31. Hattori S. Syntrophic acetate-oxidizing microbes in methanogenic environments. Microbes Environ. 2008;23:118-27.

32. Nobu M K, et al. Microbial dark matter ecogenomics reveals complex synergistic networks in a methanogenic bioreactor. ISME J. 2015;9:1710-22.

33. Chen S, X Liu, X Dong. Syntrophobacter sulfatireducens sp. nov., a novel syntrophic, propionate-oxidizing bacterium isolated from UASB reactors. Int J Syst Evol Micr. 2005;55:1319-24.

34. Wallrabenstein C, B Schink. Evidence of reversed electron transport in syntrophic butyrate or benzoate oxidation by Syntrophomonas wolfei and Syntrophus buswellii. Arch Microbiol. 1994;162:136-42.
